# MicroRNA pharmacogenomics based integrated model of miR-17-92 cluster in sorafenib resistant HCC cells reveals a strategy to forestall drug resistance

**DOI:** 10.1038/s41598-017-11943-1

**Published:** 2017-09-13

**Authors:** Faryal Mehwish Awan, Anam Naz, Ayesha Obaid, Aqsa Ikram, Amjad Ali, Jamil Ahmad, Abdul Khaliq Naveed, Hussnain Ahmed Janjua

**Affiliations:** 1 Atta-ur-Rahman School of Applied Biosciences (ASAB), National University of Sciences and Technology (NUST), H-12 Islamabad, Pakistan; 2 Research Center for Modeling and Simulation (RCMS), National University of Sciences and Technology (NUST), H-12 Islamabad, Pakistan; 3 Islamic International Medical College (IIMC), Riphah International University, Rawalpindi, Pakistan

## Abstract

Among solid tumors, hepatocellular carcinoma (HCC) emerges as a prototypical therapy-resistant tumor. Considering the emerging sorafenib resistance crisis in HCC, future studies are urgently required to overcome resistance. Recently noncoding RNAs (ncRNAs) have emerged as significant regulators in signalling pathways involved in cancer drug resistance and pharmacologically targeting these ncRNAs might be a novel stratagem to reverse drug resistance. In the current study, using a hybrid Petri net based computational model, we have investigated the harmonious effect of miR-17-92 cluster inhibitors/mimics and circular RNAs on sorafenib resistant HCC cells in order to explore potential resistance mechanisms and to identify putative targets for sorafenib-resistant HCC cells. An integrated model was developed that incorporates seven miRNAs belonging to miR-17-92 cluster (hsa-miR-17-5p, hsa-miR-17-3p, hsa-miR-19a, hsa-miR-19b, hsa-miR-18a, hsa-miR-20a and hsa-miR-92) and crosstalk of two signaling pathways (EGFR and IL-6) that are differentially regulated by these miRNAs. The mechanistic connection was proposed by the correlation between members belonging to miR-17-92 cluster and corresponding changes in the protein levels of their targets in HCC, specifically those targets that have verified importance in sorafenib resistance. Current findings uncovered potential pathway features, underlining the significance of developing modulators of this cluster to combat drug resistance in HCC.

## Introduction

Chemotherapy serves as a cornerstone in the development of current cancer therapy and is widely used in the treatment of cancer^1^. One of the ongoing challenges to increase the likelihood of success in cancer treatment is to circumvent overarching limitation of the emerging drug resistance^2^. The intricate mechanism of anticancer drug resistance has been broadly explored in recent years and has yet to be fully elucidated^3^. Thus, it is of paramount interest to overcome resistance and to encourage the research for novel chemotherapeutic approaches. The complex mechanism of chemo-resistance is a multifactorial phenomenon depending upon many factors including drug, tumor and host specific defense mechanisms^4^. One of the recently explored mechanism includes non-coding RNA (ncRNA) mediated form of drug resistance^3^. MicroRNAs (miRNAs) are short, highly conserved endogenous ncRNAs (20–23 nucleotides) that are implicated in the post-transcriptional regulation of gene expressions by base pairing with complementary mRNA. Substantial facts and evidences are looming that particular miRNA alterations are associated with tumor initiation, progression and recurrence^3^. Furthermore, miRNAs have also shown to be promising and emerging target to tackle mechanisms of chemoresistance in various cancers^5, 6^. Previously, it has been reported that varying miRNA expression profile is linked to the development of anticancer drug resistance^7^. Therefore, exploiting the therapeutic potential of miRNAs for overcoming anticancer drug resistance is of prime importance in various cancers. Hepatocellular carcinoma (HCC), as a diagnostically and therapeutically challenging tumor is notoriously refractory to standard systemic therapy because of its strong inclination towards multi-drug resistance^8^.

HCC being the most frequent histological subtype, accounts for >90% of the total liver tumor burden^9, 10^. It is among the most chemo-resistant tumors, and is the second preeminent cause of cancer-related deaths worldwide^10, 11^. As of 2016, the small molecule multikinase inhibitor, sorafenib remains the only FDA approved standard first-line systemic therapy in patients with advanced HCC^12, 13^. Regardless of encouraging results demonstrated by sorafenib, the worry for resistance is rising, as the overall survival of HCC patients after therapy is only 2–3 months longer than placebo^14^. Furthermore, low partial response rate of sorafenib is a worrisome phenomenon^15^. Multiple mechanisms including epithelial-mesenchymal transition, PI3K/Akt, JAK-STAT pathways, hypoxic microenvironment, and others, are considered to be involved in sorafenib resistance^16, 17^. At present, an in-depth mechanism by which this phenomenon occurs remains conjectural. Considering the problems related to poor effectiveness and heterogeneous individual responses to sorafenib therapy, studies regarding thorough understanding of the mechanism of sorafenib resistance are urgently required. Given the presence of complex and intricate pathways adopted by tumor cells, combination therapies are now being explored in an effort to overcome resistance.

miRNAs play a noteworthy role in pharmacogenomics by regulating the expression of genes that are imperative for drug function^18^. They are often observed to be deregulated in drug-resistant cells. Identifying possible associations between dysregulated miRNAs and drug resistance phenotype would make a significant contribution and will expedite the use of miRNA profiling to forecast drug responses. Various studies have highlighted the significance of miR-17-92 cluster in chemo-resistance. The miR-17-92 cluster encodes seven mature miRNAs (miR-17-5p, miR-17-3p, miR-18a, miR-19a, miR-19b, miR-20a, and miR-92a)^19^. This cluster has been validated as an oncogenic as well as tumor suppressive regulator. This dual role of miR-17-92 cluster is due to the diverse spectrum of targeted mRNAs as well as intricate cascade of miRNAs and their targets involved in distinct pathways^20^. Cioffi *et al*., observed down-regulation of miR-17-92 cluster in chemo-resistant cancer stem cells versus chemo-sensitive cancer stem cells. In these cancer stem cells, miR-17-92 cluster was found to target several regulators of NODAL/ACTIVIN signaling pathway having crucial role in chemo-resistance^21^. In another study conducted by Zhu *et al*., miR-17-92 cluster was observed to be up-regulated in paclitaxel resistant ovarian cancer cells^22^. Zhou *et al*., investigated the role of miR-17-92 cluster in cisplatin-resistant prostate cancer cells. The authors observed that miR-17-92 cluster activated PI3K/Akt and ERK1/2 signaling pathway, which played a central role in chemo-resistance to cisplatin^23^. Furthermore, Miles *et al*., identified potential relationship between miR-17-92 and drug resistance in burkitt lymphoma Cells. The authors observed increased chemo-sensitivity in miR-17-92 hemizygous-deleted cell lines^24^. Although our understanding of drug resistance remains incomplete, new and more innovative approaches are urgently needed to intercept this phenomenon. Further investigation and exploration of the crosstalk of miRNAs and their associated pathways may lead to future breakthroughs in the treatment of HCC and will hopefully increase our understanding of the mechanisms implicated in sorafenib resistance.

**Figure 1:**
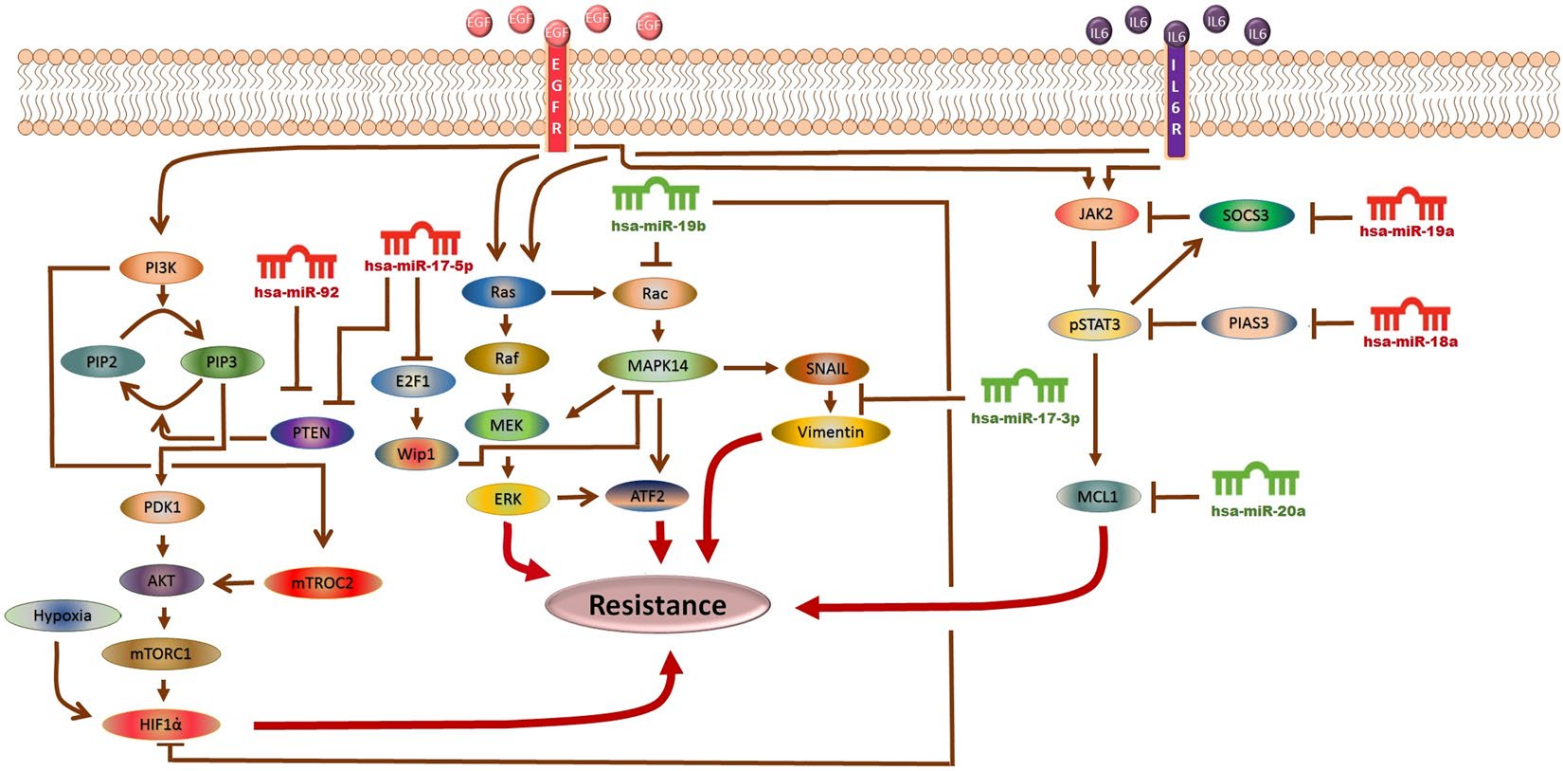
The Model Signaling Network. Hypothetical implication of miR-17-92 cluster mediated mechanism of sorafenib resistance in HCC. PTEN, Rac, HIF-1α, SOCS3, PIAS3 and MCL1 networks, downstream from EGFR/IL-6 signaling pathway were assembled from various sources. Pointed arrow represents an activation reaction, whereas a plunger indicates an inhibition reaction.

The extensive data generated via high-throughput techniques needs to be explored holistically in order to decipher the exact mechanisms involved in cancer resistance and to analyze various therapeutic options. A fundamental setback is to integrate, infer and interpret data to advance our understanding in cancer system biology^25^. The past few years have marked significant advances in the development of various methods for the representation and modeling of signal transduction pathways. These biological formalisms include ordinary differential equations (ODEs), directed graphs, Boolean networks, Bayesian networks, cellular automata, interacting state machines, stochastic equations and rule-based formalisms^26^. One of the main drawbacks in using these methods is the need for complete information on concentrations, diffusion rates, degradation rates, reaction rates and many other parameters that are difficult to measure. Usually this kind of information is not available for all biological entities; hence, estimates and approximations have to be used. Unfortunately, inaccurate estimates can often lead to significant numerical instabilities^27^. On the contrary, the strong appeal for Petri nets (PNs) lies in the fact that they do not necessitate a great deal of mathematical ability or knowledge, and do not always require precisely measured parameters before implementation. PNs are relatively non-mathematical alternatives to ODEs, and have been emerged as an efficient tool among other related methods for modeling and simulating the dynamics of biological signaling networks^27–29^. In the current study, using a hybrid Petri net (HPN)-based computational approach, we have investigated the dynamics of several signaling molecules. We constructed HPN model of the EGFR/IL-6 signaling pathway and incorporated corresponding miR-17-92-target information. We then performed an *in-silico* scrutinization of the impact of miRNAs and their corresponding mimics and inhibitors on the EGFR/IL-6 signaling pathway, representing the consequences of miR-17-92 regulatory processes on the behavior of this signaling network. By comparing experimental data with the model simulations, we have uncovered potential pathway features as well as putative targets for sorafenib resistant signaling in HCC cells. An outline of the signaling cascade used for HPN model generation is represented in Fig. 1. Current study was carried out with an objective of making correlation among miR-17-92 cluster, EGFR/IL-6 signaling pathway and sorafenib resistance through step wise simulation, validation of the proposed model and analysis of sorafenib resistant HCC cells. We expect that the current association between miR-17-92 cluster and sorafenib resistance in HCC will attract experimental research community to verify their role in resistance mechanism.

## Results and Discussion

**Figure 2:**
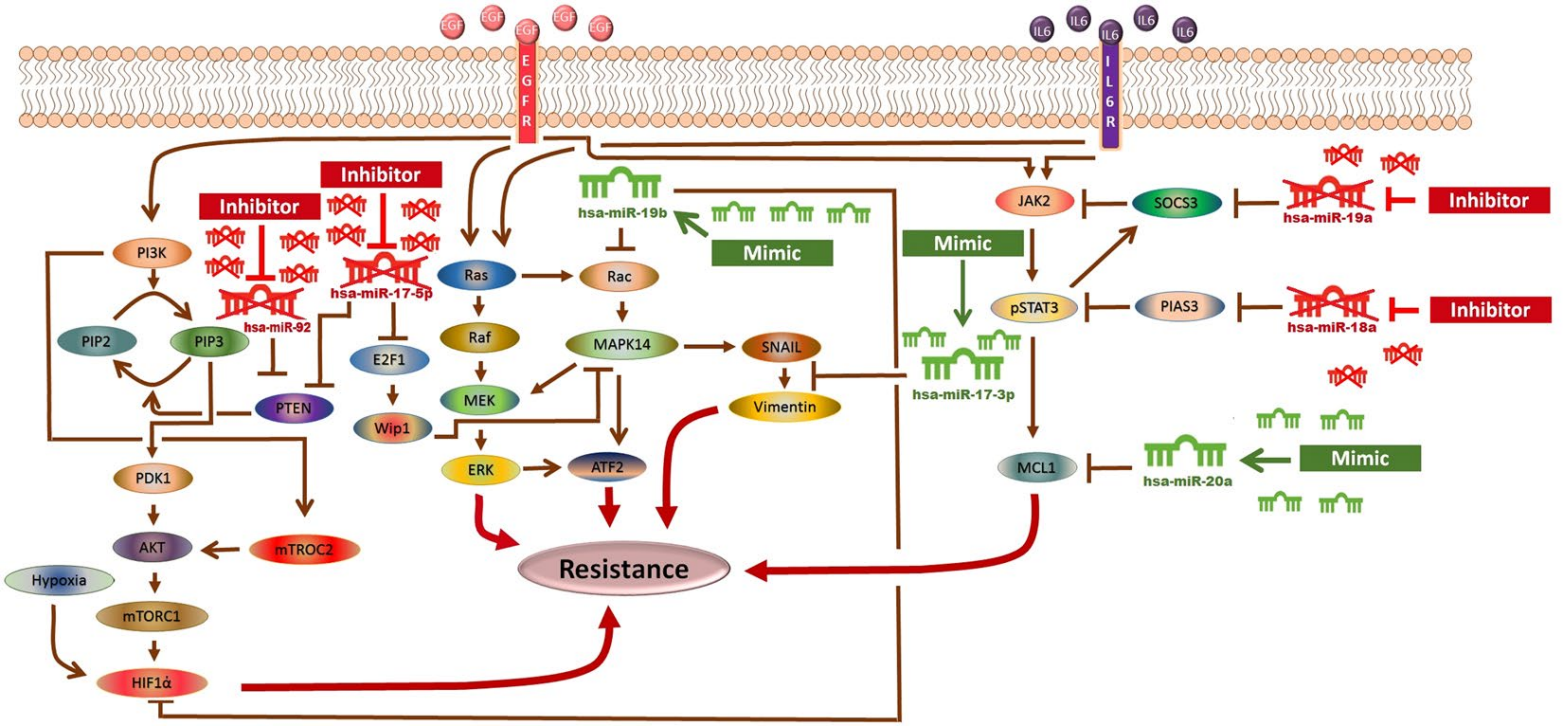
The Model Signaling Network. Hypothetical implication of combating sorafenib resistance in HCC via mimics and inhibitors of miR-17-92 cluster. PTEN, Rac, HIF-1α, SOCS3, PIAS3 and MCL1 networks, downstream from EGFR/IL-6 signaling pathway were assembled from various sources. An edge ending with pointed arrow indicates an activation reaction, while an edge ending with a plunger indicates an inhibition reaction.

The significance of an integrated model of all the essential pathways related to sorafenib resistance cannot be denied but the limitations of available kinetic data for each entity in the complex signaling cascade renders it very difficult to formulate the extensive model. Here an HPN model of combating sorafenib resistance in HCC cells has been built in a logical way via using inhibitors and mimics of miRNAs belonging to miR-17-92 cluster. An outline of combating sorafenib resistance signaling cascade employing modulators of miR-17-92 cluster which was used for HPN model generation is represented in Fig. 2. A PN model is graphically illustrated with ‘places’, representing the biological components, and ‘transitions’, describing the activity between the biological components, which can be supported by known interaction information. The availability of tokens in places signifies the relative availability of the biological component. Tokens (usually relative protein levels) are allocated on the basis of existing data from the literature. Places and transition are linked by weighted arcs that are imperative for the flow of the network.

### Establishment of an integrated model of the PI3K/Akt/mTOR/miR-17-5p/miR-17-3p/miR-19b/miR-92 pathway in sorafenib resistant HCC cells

**Figure 3:**
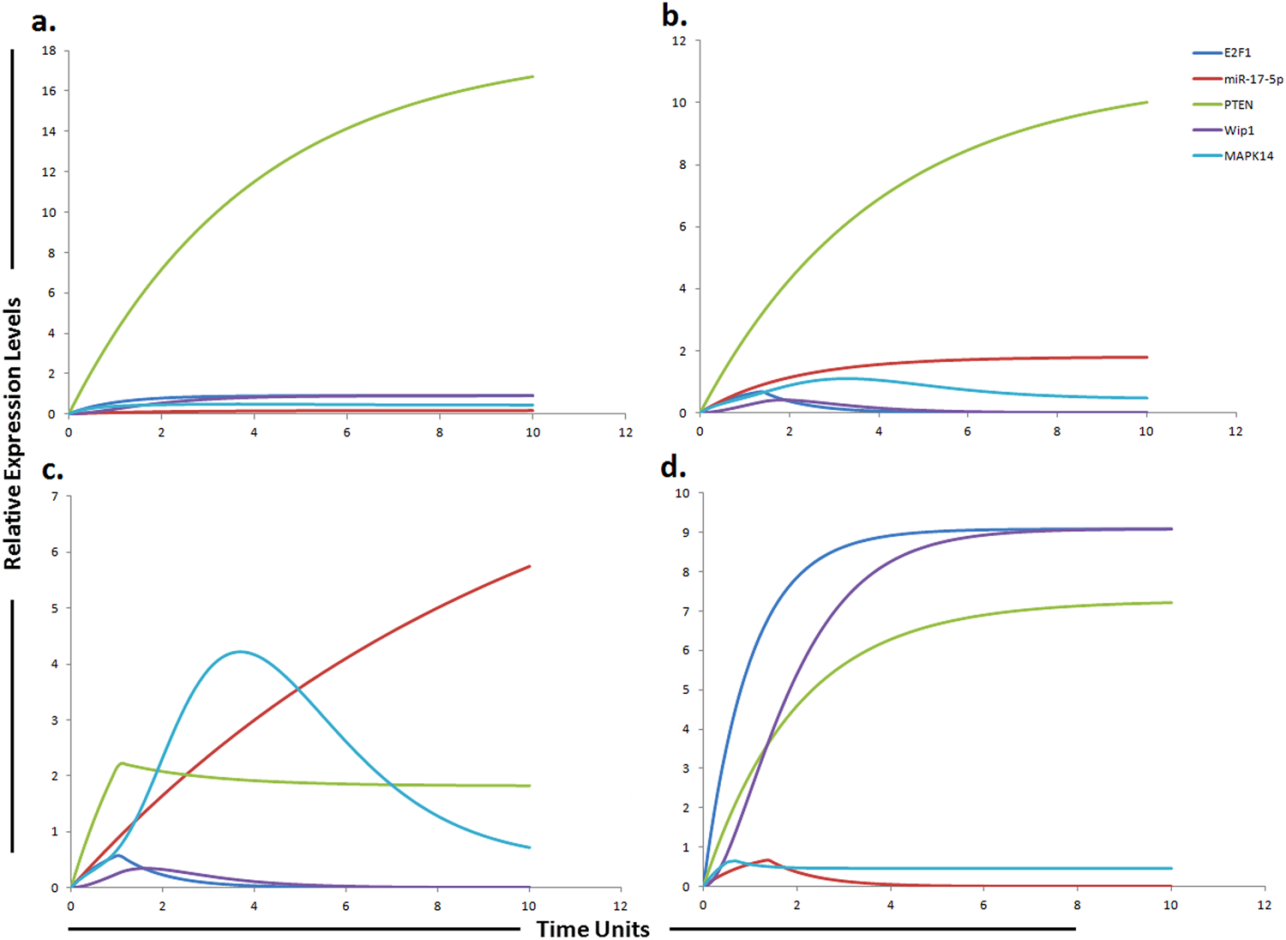
Graphical simulations illustrating main targets of miR-17-5p. Time units are represented on x-axis with relative expression levels of PTEN, E2F1, MAPK14, Wip1 and miR-17-5p on y-axis. Relative levels of PTEN, E2F1, MAPK14, Wip1 and miR-17-5p are shown in green, blue, cyan, purple and red lines respectively. (**a**) Simulation of PTEN, E2F1, Wip1, MAPK14 in normal liver cells under the influence of miR-17-5p. The kinetics of PTEN inhibition by miR-17-5p was modeled using mass action with miR-17-5p acting as a competitive inhibitor of PTEN that is known to increase sorafenib sensitivity in HCC. PTEN expression levels are known to be steady and high in normal cells^54^ as represented by green curve in the figure. Simulation is illustrating the inverse relationship between PTEN and miR-17-5p. PTEN is growing exponentially in normal state as compared to miR-17-5p which is known to inhibit the expression of PTEN. PTEN is the natural inhibitor of PI3K/Akt pathway. PTEN has also been shown to be significantly reduced in normal liver tissues compare to HCC liver tissues. (**b**) In HCC state, the level of miR-17-5p is greatly increased. Nearly two fold decrease in PTEN was found in HCC compare to the normal cells calculated from experimental results and represented by red curve. (**c**) miR-17-5p overexpression (red curve) in sorafenib resistant HCC cells reduces the level of PTEN (green curve) and increases the level of MAPK14 (blue curve). (**d**) Simulations revealed that inhibiting the effects of miR-17-5p via miR-17-5p inhibitor generates remarkable change in the expression profiles of MAPK14, PTEN, E2F1 and Wip1 with respect to the control situation. The significant phenomenon of the application of miR-17-5p inhibitor is that not only the expression levels of target protein (PTEN, E2F1), but also the indirect target proteins (Wip1, MAPK14) can be reverted. The ratio of E2F1: MAPK14: Wip1: PTEN: miR17-5p is in concordance with the published studies.

**Figure 4:**
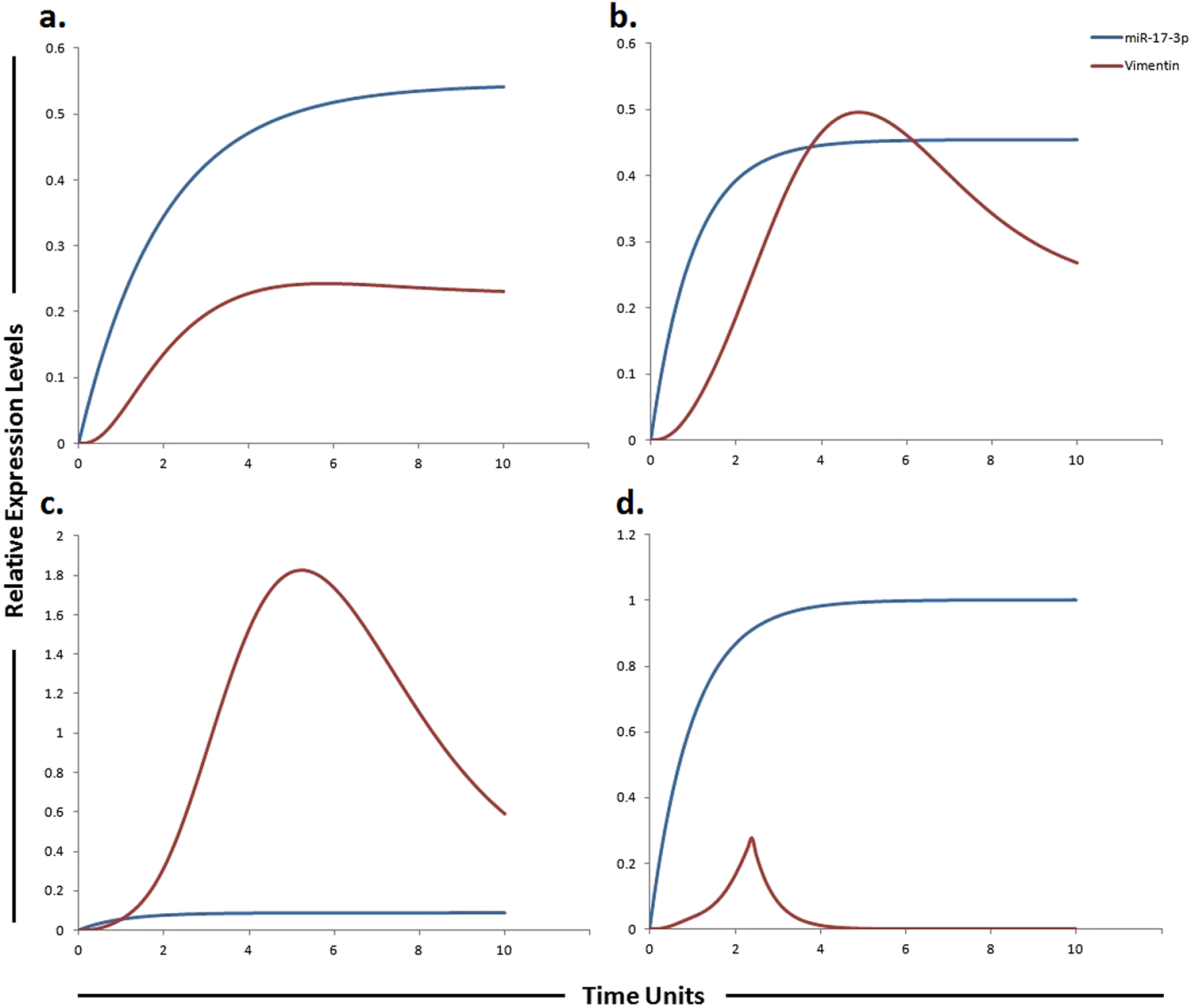
Graphical simulations illustrating main target of miR-17-3p. Time units are represented on x-axis with relative expression levels of vimentin and miR-17-3p on y-axis. Relative levels of vimentin and miR-17-3p are shown in red and blue lines respectively. (**a**) Simulation of vimentin under the influence of miR-17-3p in normal liver cells. miR-17-3p expression has been previously reported to be low in highly tumorigenic, metastatic cell lines as illustrated by blue curve in HCC (**b**) and sorafenib resistant HCC cells (**c**), but increased in cell lines which display decreased tumorigenicity as illustrated by blue curve in (**a**). The lack of miR-17-3p expression has been previously correlated with an increase in vimentin synthesis. The simulations in graph (**d**) illustrates that vimentin activity is significantly reduced after using miR-17-3p mimic. A study conducted by Shan *et al*., revealed that miR-17-3p targets vimentin in HCC cells as represented by blue curve unveiling suppressed activity level of vimentin by the up-regulation of miR-17-3p. The ratio of vimentin: miR-17-3p is in concordance with the published studies.

**Figure 5:**
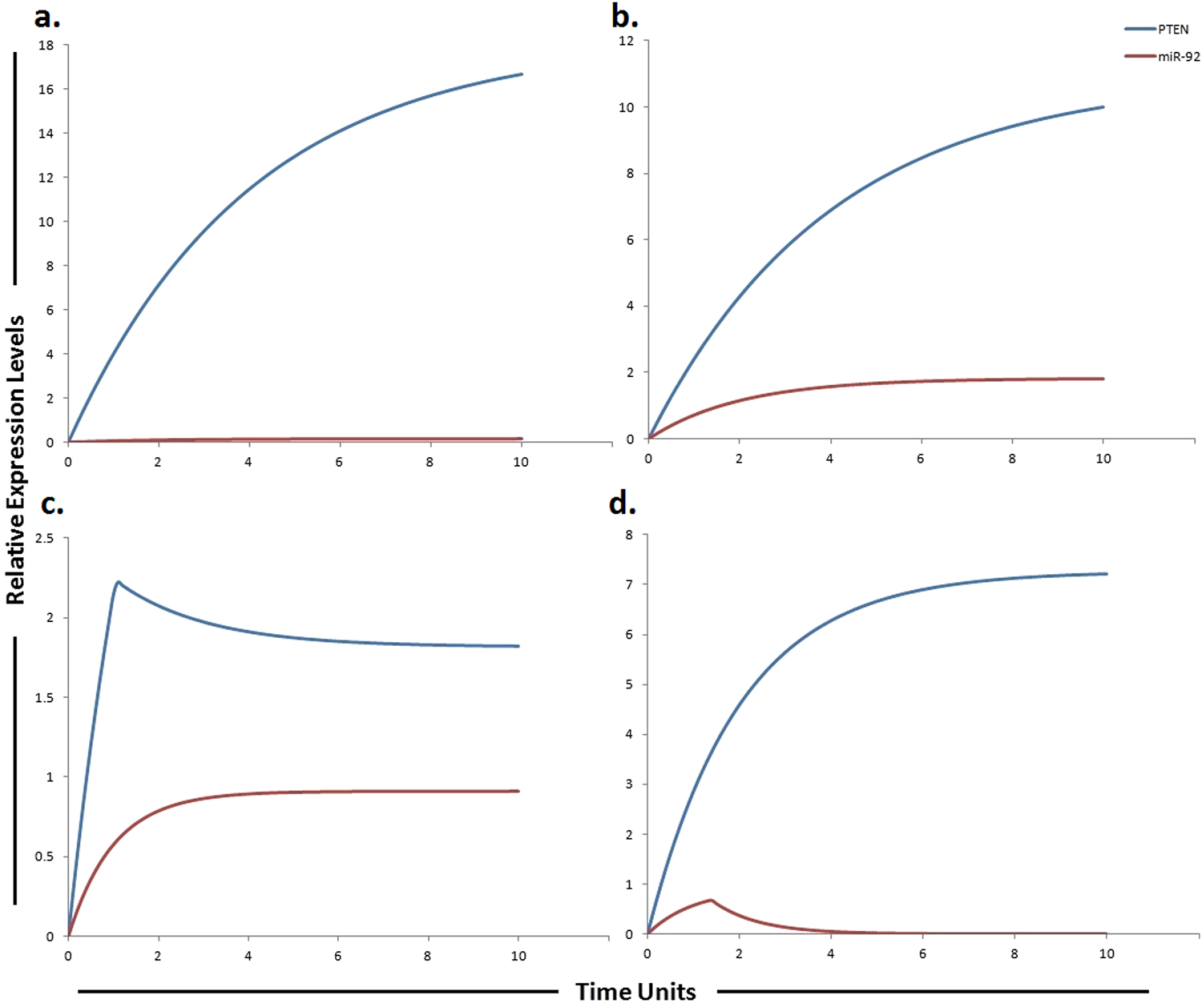
Graphical simulations illustrating main target of miR-92. Time units are represented on x-axis with relative expression levels of PTEN and miR-92 on y-axis. Relative levels of PTEN and miR-92 are shown in red and blue lines respectively. (**a**) Simulation of PTEN under the influence of miR-92. The kinetics of PTEN inhibition by miR-92a was modeled using mass action with miR-92 acting as a competitive inhibitor of PTEN that increases the sorafenib sensitivity in HCC. PTEN is the natural inhibitor of PI3K/Akt pathway and has been shown to be significantly reduced in normal liver tissues compare to HCC liver tissues, whereas, its expression levels are known to be steady and high in normal cells^54^ as represented by blue curve. Simulation is illustrating the inverse relationship between PTEN and miR-92. PTEN is growing exponentially in normal state as compared to miR-92a which is known to inhibit the expression of PTEN. (**b**) In HCC state, the level of miR-92 is greatly increased. Nearly two fold decrease in PTEN was found in HCC as compared to normal cells calculated from experimental results as represented by blue curve. (**c**) miR-92 overexpression (red curve) in sorafenib resistant HCC cells reduces the level of PTEN (blue curve). (**d**) Simulations revealed that antagonizing the effect of miR-92 via miR-92 inhibitor generates a remarkable change in the expression profile of PTEN with respect to the control situation. The ratio of PTEN: miR-92 is in concordance with the published studies.

**Figure 6:**
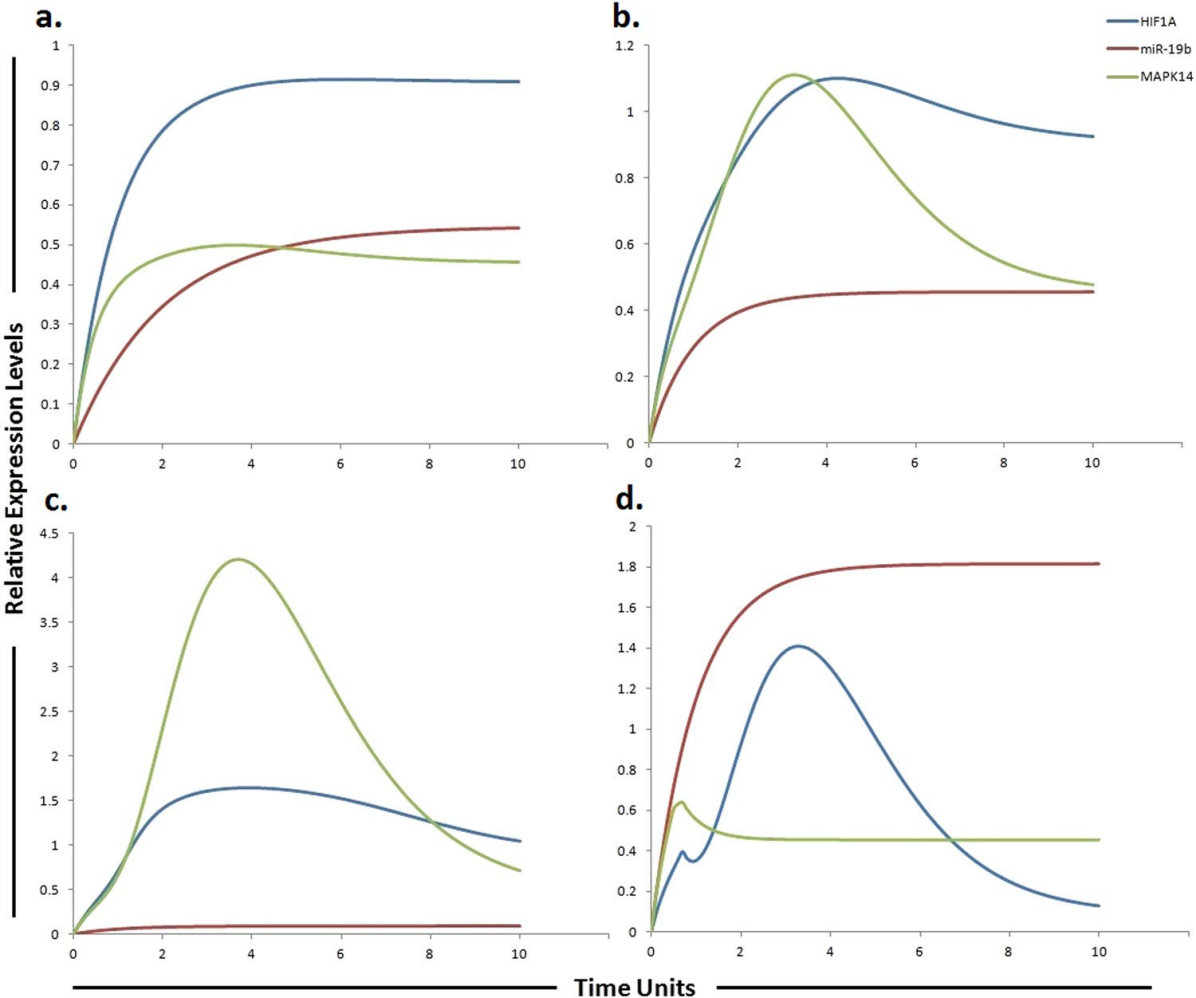
Graphical simulations illustrating HIF-1α and MAPK14 under the influence of miR-19b. Time units are represented on x-axis with relative expression levels of HIF-1α, MAPK14 and miR-19b on y-axis. Relative levels of miR-19b, MAPK14 and HIF-1α are shown in red, green and blue lines respectively. (**a**) Simulation of HIF-1α and MAPK14 in normal liver cells under the influence of miR-19b. Hung *et al*., observed increased expression of MAPK14 after suppression of miR-19b as illustrated by red curve in (**b**) and (**c**). miR-19b influences the expression of EPCAM, NDRG1, HMGB2 and HIF-1α along with MAPK14^37^. A study conducted by Liang *et al*., reported elevated level of HIF-1α in sorafenib-resistant HCC tissues (**c**) as compared to pre-treated and sorafenib-sensitive HCC tissues^57^ (**b**). Simulations in graph (**d**) illustrate that MAPK14 activity is significantly reduced after using miR-19b mimic. The ratio of HIF-1α: MAPK14: miR-19b is in concordance with the published studies.

The phosphatidylinositol 3-kinase (PI3K)/Akt/mammalian target of rapamycin (mTOR)/miR-17-5p/miR-17-3p/miR-19b/miR-92 pathway (Fig. 1) was assembled from various sources and subjected to HPN modeling. The modeled pathway was then simulated to evaluate and examine the dynamics of the system. The PI3K/Akt pathway, a major survival pathway is one of the most frequent aberrantly regulated pathways in cancers and has been observed to mediate sorafenib resistance in HCC cells^30^. This pathway is also activated in high proportion of HCC tissues and its involvement in sorafenib resistance has been attracting attention^15^. The ubiquitously expressed PI3K generates PIP3 (phosphatidyl inositol 3,4,5 tri-phosphate) by mediating phosphorylation of PIP2. PIP3 then recruits Akt and 3-phosphoinositide-dependent kinase 1 (PDK1) to the plasma membrane where PDK1 activates Akt and mTORC2. PTEN dephosphorylates PIP3 back to PIP2. Chen *et al*., observed high pAkt expression and low PTEN expression in sorafenib resistant cells compared with the parental cells^30^ (Table 1). Furthermore, sorafenib resistance was shown to be reversed by Akt knockdown and Akt inhibitor, thus validating the contribution of activated PI3K/Akt pathway in sorafenib resistance^30^. PTEN is the natural inhibitor of PI3K/Akt pathway. In our proposed HPN model, we used miR-17-5p inhibitor to target this pathway as miR-17-5p has been shown to target PTEN in HCC cells^31^ suggesting a possible relation between miR-17-5p and sorafenib resistance in HCC (Table 2). Simulation results revealed that its inhibitor can significantly upregulate PTEN expression (Fig. 3). Furthermore, miR-17-5p indirectly activates p38 MAPK (MAPK14) pathway through E2F1^32, 33^. Previous studies revealed that MAPK14 blockade is a promising strategy to overcoming sorafenib resistance in HCC^34^. Based on these findings, we postulated that miR-17-5p inhibitor can increase the level of PTEN and decrease the level of MAPK14 whose expressions are observed to be deregulated in sorafenib resistant HCC cells. Simulation results revealed that the concentrations of miR-17-5p target proteins (PTEN and MAPK14) are inversely associated with the miR-17-5p gene expression levels (Fig. 3). Another important factor in sorafenib resistant cells is vimentin, a mesenchymal marker and previous studies have shown that Sorafenib resistant HCC cells displayed an increased expression of Vimentin^35^. miR-17-3p, a member of miR-17-92 cluster has been shown to target vimentin in HCC cells^31^ (Table 3). We proposed that its mimic can reduce the level of vimentin in sorafenib resistant HCC cells. Simulation results revealed exponential increase in vimentin after using the miR-17-3p mimic (Fig. 4). Furthermore, miR-92 expression has been shown to be inversely related with PTEN in HCC and studies revealed that miR-92 might inhibit its expression^36^ unveiling its potential role in sorafenib resistance. Therefore, we hypothesized that miR-92 inhibitor might increase the level of PTEN in sorafenib resistant HCC cells. Simulation results revealed that the PTEN activity is increased after the use of miR-92a inhibitor which antagonizes the inhibition effect of miR-92a (Fig. 5). miR-19b, another member of miR-17-92 cluster has been observed to influence the expression of MAPK14^37^. Hung *et al*., observed overexpression of MAPK14 after suppression of miR-19b revealing that its mimic might play an important role in overcoming sorafenib resistance (Fig. 6).

**Table 1:** Experimentally derived markings (fold changes) in HCC and sorafenib resistant HCC as compared to normal cells used in the simulations.

Signaling molecules	HCC	References	Sorafenib resistant HCC	References
EGFR	1.98 ↑	80	10.82 ↑	39
PTEN	2 ↓	81	5.44 ↓	30
IL-6	25 ↑	38	4.5 ↑	38
JAK-2	11 ↑	82	1.83↑	40
STAT3	8 ↑	82	5.85 ↑	40
MCL1	3.73 ↑	83	2.67 ↑	40

**Table 2:** Experimentally derived markings (fold changes) in miR-17-5p overexpressing and in anti-miR-17-5p expressing HCC cells used in the simulations.

Observations	Experimental Findings (Fold Change)	References
**miR-17-5p overexpressing cells**		
miR-17-5p	6 ↑	52
p38MAPK	7 ↑	52
E2F1	0.70 ↓	52
Wip1	0.69 ↓	52
miR-17-5p	0.4↑	31
PTEN	0.85 ↓	31
**Anti-miR-17-5p expressing cells**		
miR-17-5p	0.6 ↓	31
PTEN	2.06 ↑	31
PAkt	1.71 ↑	31

**Table 3:** Experimentally derived markings (fold changes) in miR-17-3p overexpressing and in anti-miR-17-3p expressing HCC cells used in the simulations.

Observations	Experimental Findings (Fold Change)	References
**miR-17-3p over expressing cells**		
miR-17-3p	0.6↑	31
Vimentin	0.83 ↓	31
**Anti-miR-17-3p expressing cells**		
miR-17-3p	0.8 ↓	31
Vimentin	2.57 ↑	31

### Establishment of an integrated model of the IL-6/STAT3/miR-19a/miR-18a/miR-20a pathway in sorafenib resistant HCC cells

**Figure 7:**
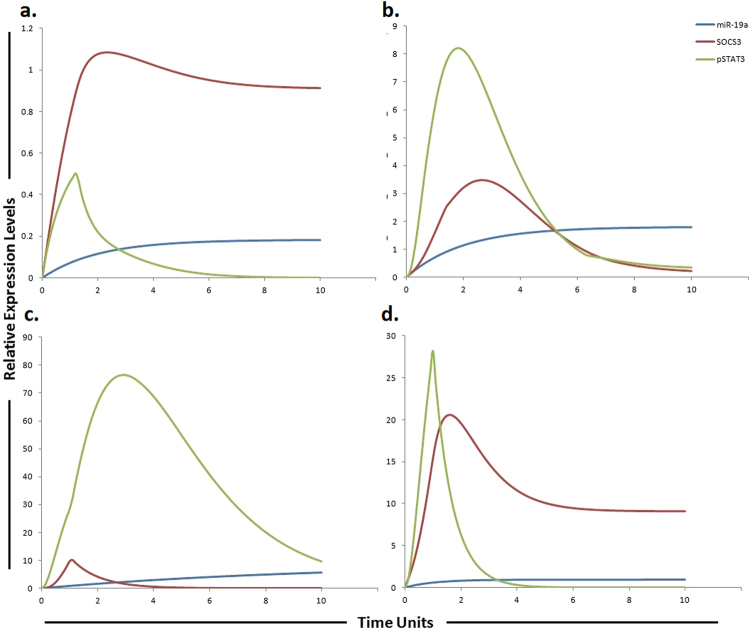
Graphical simulations of STAT3 and SOCS3 under the influence of miR-19a and their dynamic behavior with time. Time units are represented on x-axis with relative expression levels of SOCS3, pSTAT3 and miR-19a on y-axis. Relative levels of SOCS3, pSTAT3 and miR-19a are shown in red, green and blue lines respectively. (**a**) Under physiological conditions in normal cells, the activation of STAT3 protein is rapid and transient because of negative regulation by proteins such as SOCS3 as illustrated in simulations. SOCS3 play a fundamental role to keep STAT3 activity at low levels in normal conditions. (**b**) miR-19a is known to inhibit SOCS3 which is a negative regulator of STAT3 as illustrated in simulations. (**c**) High expression of miR-19a promotes sorafenib resistance through repression of SOCS3 and upregulation of STAT3 in STAT3-SOCS3 negative feedback loop. (**d**) The simulations illustrates that STAT3 activity is significantly reduced (green curve) and SOCS3 activity is significantly enhanced (red curve) as the concentration of miR-19a decreases (blue curve) after using miR-19a inhibitor. The ratio of SOCS3: pSTAT3: miR-19a is in concordance with the published studies.

**Figure 8:**
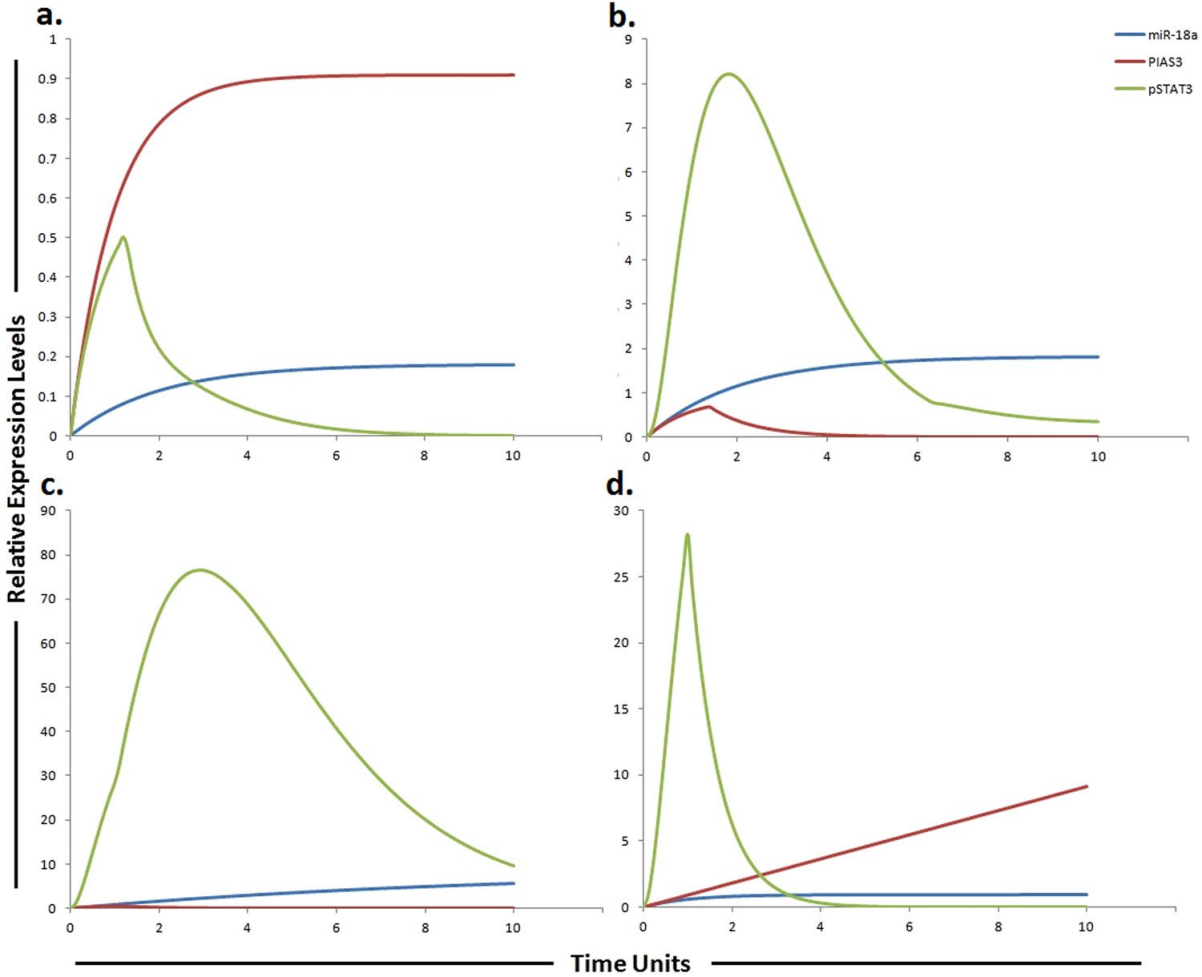
Graphical simulations of PIAS3 and STAT3 under the influence of miR-18a and their dynamic behavior with time. Time units are represented on x-axis with relative expression levels of PIAS3, pSTAT3 and miR-18a on y-axis. Relative levels of PIAS3, pSTAT3 and miR-18a are shown in red, green and blue lines respectively. (**a**) Under physiological conditions in normal cells, the activation of STAT3 protein is rapid and transient because of negative regulation by proteins such as PIASS3 as illustrated in simulations. (**b**) miR-18a is known to inhibit PIAS3 which is a negative regulator of STAT3 as illustrated in simulations. (**c**) High expression of miR-18a promotes sorafenib resistance through repression of PIAS3 and upregulation of STAT3 in STAT3-SOCS3/ PIAS3 negative feedback loop. (**d**) The simulations illustrates that STAT3 activity is significantly reduced (green curve) and PIAS3 activity is significantly enhanced (red curve) as the concentration of miR-18a decreases (blue curve) after using miR-18a inhibitor. The ratio of PIAS3: pSTAT3: miR-18a is in concordance with the published studies.

**Figure 9:**
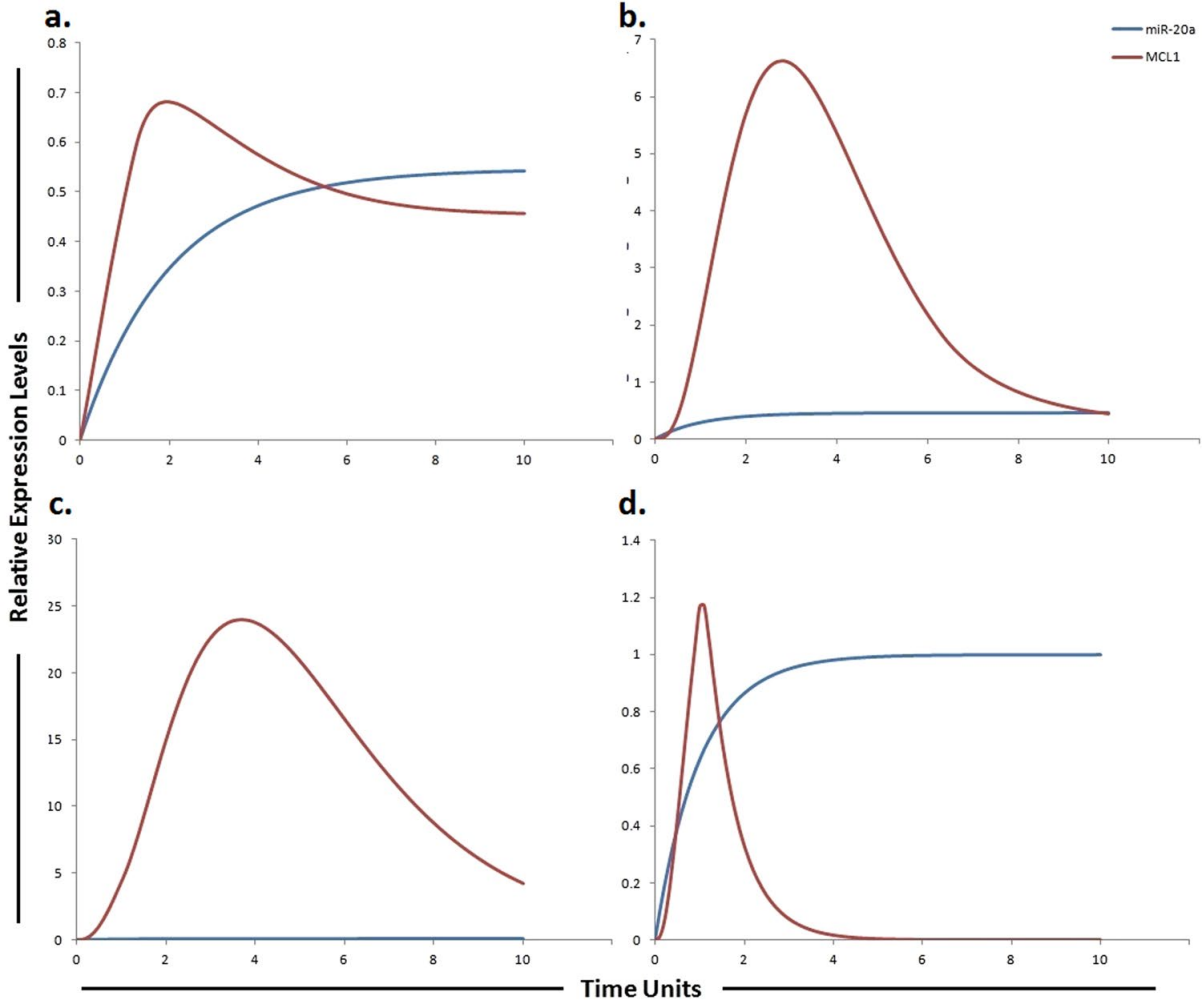
Graphical simulations illustrating MCL1 under the influence of miR-20a. Time units are represented on x-axis with relative expression levels of MCL1 and miR-20a on y-axis. Relative levels of MCL1 and miR-20a are shown in red and blue lines respectively. (**a**) miR-20a has been previously shown to directly target MCL1 resulting in its decreased protein level as illustrated by red curve. (**b**) Decrease expression of miR-20a promotes HCC cell proliferation and predicts poor survival of HCC as illustrated by increased MCL1 expression (red curve). (**c**) 3 fold increase in MCL1 levels were found in sorafenib resistant cells as compared to sorafenib sensitive HCC cells as illustrated by red curve. Simulations in graph (**d**) illustrate that MCL1 activity is significantly reduced after using miR-20a mimic. Simulation results of our designed HPN model showed the respective fold increments in MCL1 level. Furthermore, simulations revealed that miR-20a mimic significantly reduced the level of MCL1 whose expression is observed to be increased in sorafenib resistant HCC cells. The ratio of MCL1: miR-20a is in concordance with the published studies.

The IL-6/STAT3/miR-19a/miR-18a/miR-20a pathway was assembled from various sources and subjected to HPN modeling. The tokens were adjusted according to the literature findings from various experimental studies performed individually. IL-6, a pleiotropic cytokine, signals through gp130/Janus kinases (JAKs)/STAT pathway. IL-6-induced JAK family members activate three major pathways, STAT3, PI3K and MAPK^38^. Endogenous IL-6 levels have been reported to be higher in SNU-449 cells which are considered to be sorafenib resistant cells^38, 39^. Binding of IL-6 to its receptor IL-6R, results in the formation of IL-6/IL-6R complex. Mass action kinetics was used to model the binding and dissociation of this complex. The IL-6/IL-6R complex activates STAT3 causing STAT3 phosphorylation via activation of JAK2. Significant activation of STAT3 has been reported in sorafenib resistant cells, signifying that targeting STAT3 may represent an attractive strategy to surmount sorafenib resistance^40^ (Table 1). Once activated, phosphorylated STAT3 (pSTAT3) up-regulates the expression of its target gene MCL1. Suppressor of cytokine signaling (SOCS) proteins negatively regulates STAT3 by inhibiting its activation through JAK Suppressor of cytokine signaling 3 (SOCS3)^41^. SOCS3 act as a competitive inhibitor that mainly targets the gp130 protein to inhibit STAT3 activation. Members of the SOCS family are the main physiological negative regulators of IL-6 signaling. Croker *et al*., reported a prolonged activation of STAT3 after conditional deletion of SOCS3 in liver and macrophages^42^. miR-19a, a member of miR-17-92 cluster has been reported to augment the JAK-STAT signal transduction via control of SOCS3 expression. Upon transfection with miR-19a, Collins *et al*., reported significant down regulation of SOCS3, a JAK-STAT pathway inhibitor^43^. Based on these findings, we postulated that miR-19a inhibitor can thus increase the level of SOCS3, ultimately reducing the STAT3 level. Simulation results revealed that the SOCS3 activity is increased after the use of miR-19a inhibitor which antagonizes the inhibition effect of miR-19a (Fig. 7). miR-18a, another member of miR-17-92 cluster promotes phosphorylation and expression of STAT3 by targeting PIAS3 (a repressor of STAT3 activity) in human hepatocytes^44^. Thus we proposed that miR-18a inhibitor can reduce the level of STAT3 by increasing the level of PIAS3 (an inhibitor of STAT3) which ultimately will help to overcome sorafenib resistance. We tested our hypothesis using HPN, whether inhibitors of miR-19a and miR-18a are sufficient to augment the level of SOCS3 and PIAS3 levels. Simulations showed that both SOCS3 and PIAS3 levels were significantly increased, when inhibitors were applied (Figs 7 and 8). Our model approximates the upregulation of pSTAT3 by miR-18a and miR-19a along with the downregulation of pSTAT3 by inhibitors of miR-18a and miR-19a using mass action kinetics. The model was further extended to include additional signaling pathways in order to accomplish a decrease in sorafenib resistance. Study conducted by Tai *et al*., showed higher level of MCL1 expression in sorafenib resistant cell line which is a downstream signaling molecule of pSTAT3^40^. miR-20a directly targeted MCL1 and reduced its protein level in HCC cells^45^. We hypothesized that miR-20a mimic can reduce the level of MCL1 whose expression is observed to be increased in sorafenib resistant HCC cells. Simulation results showed that a miR-20a mimic significantly reduced the expression level of MCL1 (Table 1 and Fig. 9).

### Analysis of predictive ability of the miR-17-92/EGFR/IL-6 signaling HPN model

After establishing the miR-17-92 mediated sorafenib resistance model, we set out to validate its predictive value. We showed the predictive ability of our model by comparing the *in-silico* simulation data with the experimental results which reached high accuracy (Table 4). Our proposed model and the results of targeted experiments reflect the behavior of the proteins correctly when subjected to specific perturbations unveiling the soundness of the model. The abstract nature of our model and the ease of its construction make it a very good candidate for modeling integrated networks from qualitative data. Therefore, we conclude that our model is able to predict cellular responses to altered miRNA levels. We can effortlessly test the concentration of any molecules in the system and bring forth any effect on the system as the state of each element in our model can be easily obtained. Furthermore, a biologist can use and extend our model by introducing perturbations in the system which can be done by the knockout of genes, the inhibition of proteins, changes in environmental factors etc. These perturbations can be introduced by addition of arcs, removal of arcs or introduction of inhibitory edges. From each new initial state, another sequence of states and possibly other terminal states can be observed depending upon the nature and type of experiment being performed. We further extended our proposed model by studying the effect of ERK inhibitor on sorafenib resistant HCC cells. Recently, Regorafenib, a multi-kinase inhibitor has been reported to inhibit the growth of sorafenib-resistant HCC cells by suppressing the ERK signaling pathway^46^. The authors suggested that sorafenib-resistance might be related to the acquisition of resistance against ERK signaling pathway inhibition mediated by sorafenib. Interestingly, regorafenib treatment was shown to suppress the tumor growth of sorafenib-resistant Huh7 and Huh1 clones. The efficacy of regorafenib on the sorafenib-resistant cells implies its potential utility as a second line treatment in HCC patients who showed progression following initial sorafenib treatment. Therefore, a perturbation experiment was designed to extend our model and scrutinize the role of ERK inhibitor on sorafenib resistant HCC cells in order to determine whether ERK inhibition has any impact on sorafenib resistance or not. We modeled the activity of ERK inhibitor by introducing the inhibitor arc in the sorafenib resistant pathway. Simulation results revealed that ERK inhibition has the ability to impact sorafenib resistance (Figure S1). Currently, the model describes only the dynamics of members belonging to miR-17-92 cluster, but its setup allows future extensions to include more miRNAs and their target molecules to depict a more comprehensive analysis, with a better predicting power in terms of capturing both the general dynamics well as the specific profile changes of key intermediate species.

**Table 4:** Summary of the effect of perturbations reported by experimental and simulated methods.

Molecules	HCC		Sorafenib resistant HCC	
	Experiment	Simulation	Experiment	Simulation
EGFR	↑	↑	↑↑	↑↑
PTEN	↓	↓	↓↓	↓↓
IL-6	↑	↑	↑↑	↑↑
JAK-2	↑	↑	↑↑	↑↑
STAT3	↑	↑	↑↑	↑↑
MCL1	↑	↑	↑↑	↑↑
p-ERK	↑	↑	↑↑	↑↑
p-AKT (S473)	↑	↑	↑↑	↑↑
p-AKT(T-308)	↑	↑	↑↑	↑↑
Vimentin	↑	↑	↑↑	↑↑
PI3K	↑	↑	↑↑	↑↑

The up-arrow (↑) specifies a rise in the level of the respective protein and the down-arrow (↓) illustrates that a decrease occurred. ImageJ software was used to calculate values in the e*xperiment* column.

### Modeling the impact of miR-17-5p and its targets PTEN and E2F1 on sorafenib resistance

miRNA inhibitory effect can lead to normalizing the expression levels of its numerous target proteins, which might result in recovery of a cell from a resistant state to a sensitive state. We therefore wanted to scrutinize the effect of miR-17-5p and its inhibitor on its direct (PTEN and E2F1) as well as indirect targets (Wip1 and MAPK14) within sorafenib resistant EGFR/IL-6 signaling cascade based on the evidences provided by various studies. PTEN, a gatekeeper tumor suppressor gene has been reported to exert its tumor suppressing function via negatively regulating PI3K/Akt cell survival signaling pathway by dephosphorylating the D3 position of PIP3^47, 48^. Activated PI3K/Akt pathway has been observed to play a significant role in promoting acquired resistance to sorafenib in HCC^30, 49^. Furthermore, PTEN has also been shown to be significantly reduced in normal liver tissues compare to HCC liver tissues^48^. Huang *et al*., reported significantly shorter overall survival in HCC patients with decreased expression of PTEN^50^. In another study, PTEN was reported to significantly affect sensitivity of HepG2 cells towards sorafenib, and the authors observed that cell killing effect of sorafenib was significantly enhanced when PTEN was overexpressed^51^. Previously miR-17-5p is known to target PTEN in HCC cells^31^ suggesting a possible link between miR-17-5p and sorafenib resistance in HCC. The authors suggested that combination of sorafenib and PTEN overexpression will likely improve therapeutic options for HCC. The authors observed increased PTEN expression after transfection of cells with the miR-17-5p inhibitor^31^. Similarly, miR-17-5p has also been reported to indirectly activate p38 MAPK (MAPK14) pathway through E2F1. E2F1 is known to activate Wip1 (a p38 MAPK inhibitor). Both Wip1 and E2F1 levels have been shown to be decreased in miR-17-5p overexpressing HCC cells. Ectopic expression of Wip1 resulted in inactivation of the p38 MAPK pathway in miR-17-5p-overexpressing HCC cells^52^. Rudalska *et al*., performed *in-vivo* RNA interference screening in transposon-based sorafenib resistant mouse model to find out important determinants of resistance in liver cancer. The authors reported that silencing MAPK14 restored sorafenib sensitivity and strongly augmented its therapeutic efficacy. They observed noticeable p-MAPK14 clonal expression in biopsies of patients getting sorafenib therapy. They suggested that the stimulation of the MAPK14 signaling cascade signifies an important mechanism of sorafenib resistance in liver cancers and MAPK14 blockade is a hopeful approach to overcome sorafenib resistance. Exposure of HCC cells to MAPK14 inhibitors significantly reduced the levels of phosphorylated Hsp27, a verified downstream target of MAPK14. The authors also observed that long-term exposure to sorafenib resulted in strong induction of p-ERK and p-Mek levels^34^. As miR-17-5p considerably stimulate the MAPK pathway and also augment the phosphorylation of Hsp27 in HCC cells^32, 33^ therefore we hypothesize that by using miR-17-5p inhibitor we can enhance sensitivity to sorafenib by blocking MAPK14, as study conducted by rudalska *et al*., suggests that sorafenib in combination with MAPK14 inhibitor is a hopeful strategy to surmount therapy resistance of HCC. Furthermore, elevated levels of miR-17-5p have also been correlated with overall low survival rates in HCC^53^. We therefore chose to test the impact of miR-17-5p inhibitor and performed simulations. By decreasing the level of miR-17-5p in our proposed model using miR-17-5p inhibitor, the expressions of its direct target proteins (PTEN and E2F1) as well as many downstream proteins were affected, signifying a direct and indirect effect on EGFR signaling pathway. The graphical simulations revealed that the increased expression of mir-17-5p corresponds with a reduced level of target protein’s expression that is PTEN and E2F1. PTEN expression levels are known to be steady and high in normal cells^54^ as illustrated in Fig. 3. Application of miR-17-5p inhibitor seems to overcome miR-17-5p induced down-regulation of PTEN. Keeping all these in relevance, we proposed a hypothetical model suggesting that if miR-17-5p regulates the expression of the PTEN and MAPK14, which are known critical factors in sorafenib sensitivity, then this correlation may have an important contribution in the development of resistance to sorafenib and if we use miR-17-5p inhibitor we can enhance sensitivity to sorafenib or can enhance the time period required for the development of sorafenib resistance.

As miR-17-92 cluster is considered to be highly oncogenic in nature therefore we kept their level at minimum in HPN model of normal liver cells. ImageJ was used to calculate fold changes of respective proteins by using western blot images of above mentioned studies. Tokens used in HPN model were based on experimental fold changes. Nearly two fold decrease in PTEN was found in HCC as compared to normal cells calculated from experimental results. Furthermore, approximately 6 fold reduction of PTEN was found in sorafenib resistant HCC cells as compared to sorafenib sensitive HCC cells. Antagonizing the effect of miR-17-5p via miR-17-5p inhibitor generates a remarkable change in the expression profiles of MAPK14, PTEN, E2F1 and Wip1 with respect to the control situation. Figure 3 illustrates the dynamical behavior of each entity that can be seen clearly through simulation graph plotted relative to the expression level of entities with respect to time. Results of our designed HPN came out to be in agreement with experimental results. Simulations of miR-17-5p, MAPK14, PTEN, E2F1 and Wip1 in normal, HCC, sorafenib resistant HCC and sorafenib resistant HCC cells after the use of miR-17-5p inhibitor are shown in Fig. 3.

### Simulating the effect of miR-17-3p and its target vimentin in sorafenib resistance

Vimentin is a mesenchymal marker and previous studies have shown that Sorafenib resistant HCC cells displayed an increased expression of vimentin^35^. Tissue microarray revealed that high level of vimentin was considerably related with HCC metastasis (P < 0.01), suggesting that elevated vimentin expression may play a crucial role in HCC metastasis^55^. Another study revealed that miR-17-3p targets vimentin in HCC cells^31^ suggesting that the increment of a miR-17-3p level in the sorafenib resistant HCC cells may increase their sensitivity to sorafenib. Based on these findings, we suggested that a miR-17-3p mimic can enhance sensitivity to sorafenib or can enhance the time period required for development of sorafenib resistance by reducing the level of vimentin. Previously a study performed by Huang *et al*., showed that a LncRNA-Dreh could bind to vimentin and reduce its expression, which ultimately inhibits HCC metastasis suggesting that targeting vimentin can help in tumor suppression^56^. After calculating fold changes of respective proteins using ImageJ software, tokens were assigned to HPN model. The simulations shown in Fig. 4 represent the entities (vimentin and miR-17-3p) involved in sorafenib resistance and their dynamic behavior with time. The simulations in graph illustrates that vimentin activity is significantly reduced after using miR-17-3p mimic. Introducing miR-17-3p mimic in sorafenib resistant HCC cells resulted in down-regulation of vimentin. Results of our designed HPN came out to be in agreement with experimental results. Simulations of miR-17-3p and vimentin in normal, HCC, sorafenib resistant HCC and sorafenib resistant HCC cells after the use of miR-17-3p mimic are shown in Fig. 4.

### Proposed implication of miR-92 and its target PTEN in sorafenib resistance

Study conducted by Zhao *et al*., observed inverse correlation between tumor suppressor gene PTEN and miR-92 in HCC clinical tissues. The author’s conclusion also indicates that miR-92 may induce tumorigenesis by inhibiting the expression of PTEN^36^. After calculating fold changes of respective proteins using ImageJ software, tokens were assigned to HPN model. Nearly two fold decrease in PTEN was found in HCC as compared to normal cells calculated from experimental results. Furthermore, approximately 6 fold reduction of PTEN was found in sorafenib resistant HCC cells as compared to sorafenib sensitive HCC cells. Results of our designed HPN came out to be in agreement with experimental results. Figure 5 illustrates the dynamical behavior of each entity plotted relative to the expression level of entities with respect to time. The red line represents the relative level of miR-92 and the blue line represents the relative level PTEN. PTEN expression levels are known to be steady and high in normal cells as illustrated in Fig. 5a. Simulation is illustrating the inverse relationship between PTEN and miR-92. As miR-92 is overexpressed in HCC compared to normal cells therefore we kept its level very low in normal cells. Expression level PTEN was observed to be increased when miR-92 inhibitor was used. Simulations revealed that miR-92 inhibitor increased the level of PTEN whose expression is observed to be decreased in sorafenib resistant HCC cells (Fig. 5).

### Proposed implication of miR-19b and its targets MAPK14 and HIF-1α in sorafenib resistance

Rudalska *et al*., performed *in-vivo* RNA interference screening in transposon-based sorafenib resistant mouse model to find out important determinants of resistance in liver cancer. The authors reported that silencing MAPK14 restored sorafenib sensitivity and strongly augmented its therapeutic efficacy. They observed noticeable p-MAPK14 clonal expression in biopsies of patients getting sorafenib therapy. They suggested that the stimulation of the MAPK14 pathway signifies an important mechanism of sorafenib resistance in liver cancers and MAPK14 blockade is a hopeful approach to overcome sorafenib resistance. miR-19b, another member of miR-17-92 cluster has been observed to influence the expression of MAPK14. Hung *et al*., observed increased expression of MAPK14 after suppression of miR-19b. miR-19b was also shown to influence the expression of EPCAM, NDRG1, HMGB2 and HIF-1α along with MAPK14^37^. A study conducted by Liang *et al*., reported elevated level of HIF-1α in sorafenib-resistant HCC tissues as compared to pre-treated and sorafenib-sensitive HCC tissues^57^. Based on these findings, we postulated that a miR-19b mimic can enhance sensitivity to sorafenib or can enhance the time period required for development of sorafenib resistance via reducing the level of MAPK14 and HIF-1α. After calculating fold changes of respective proteins using ImageJ software, tokens were assigned to HPN model. Figure 6 presents the simulation results of miR-19b, HIF-1α and MAPK14, illustrating the relative levels of miR-19b, HIF-1α and MAPK14 in normal cells, HCC cells, sorafenib resistant HCC cells and in sorafenib resistant cells after augmenting the miR-19b impact by miR-19b mimic. The red line represents the relative level of miR-19b, the blue line represents the relative level of HIF-1α and the green line represents the relative level of MAPK14. Results of our designed HPN came out to be in agreement with experimental results. Simulations of miR-19b, HIF-1α and MAPK14 in normal, HCC, sorafenib resistant HCC and sorafenib resistant HCC cells after the use of miR-19b mimic are shown in Fig. 6.

### Predicting the consequences of miR-19a and its target SOCS3 on sorafenib resistance

Due to significant activation, STAT3 is considered as a key factor in sorafenib resistance. Furthermore, the expression of downstream effectors of STAT3, such as cyclin D1 and MCL1 are also found to be increased in resistant cells. Therefore JAK/STAT3 signaling pathway is considered as an important and critical modulator of the efficacy of sorafenib and targeting this pathway may be a valuable approach to surmount sorafenib resistance in HCC^40^. SOCS3 a verified target of miR-19a, negatively regulate JAK-STAT pathway. Upon transfection of miR-19a, SOCS3, a JAK-STAT pathway inhibitor, was significantly reduced (p = 0.0010)^43^. Keeping above studies in relevance, we hypothesized that a miR-19a inhibitor can enhance sensitivity to sorafenib or can enhance the time period required for the development of sorafenib resistance. After calculating fold changes of respective proteins using ImageJ software, tokens were assigned to HPN model. Figure 7 presents the simulation results of miR-19a, SOCS3 and STAT3, illustrating the relative levels of miR-19a, SOCS3 and STAT3 in normal cells, HCC cells, sorafenib resistant HCC cells and in sorafenib resistant cells after inhibition by miR-19a inhibitor. The red line represents the relative level of SOCS3, the blue line represents the relative level of miR-19a and the green line represents the relative level of STAT3. Results of our designed HPN came out to be in agreement with experimental results. Simulations of miR-19a, SOCS3 and pSTAT3 in normal, HCC, sorafenib resistant HCC and sorafenib resistant HCC cells after the use of miR-19a inhibitor are shown in Fig. 7.

### Predicting the impact of miR-18a and its target PIAS3 on sorafenib resistance

Due to significant activation, STAT3 is considered as a key factor in sorafenib resistance. Furthermore, the expression of downstream effectors of STAT3, such as cyclin D1 and MCL1 are also found to be increased in resistant cells. Therefore JAK/STAT3 signaling pathway is considered as an important and critical modulator of the efficacy of sorafenib and targeting this pathway may be a valuable approach to surmount sorafenib resistance in HCC^40^. PIAS3 (a repressor of STAT3 activity) is a verified target of miR-18a. In human hepatocytes, miR-18a increases the expression of STAT3 by targeting PIAS3^44^. Based on these findings we hypothesized that miR-18a inhibitor can reduce the level of STAT3 by increasing the level of PIAS3 (an inhibitor of STAT3) which ultimately will help to overcome sorafenib resistance. After calculating fold changes of respective proteins using ImageJ software, tokens were assigned to HPN model. The simulation shown in Fig. 8 represents the entities (miR-18a, PIAS3 and STAT3) involved in sorafenib resistance and their dynamic behavior with time. The simulations in graph illustrates that STAT3 activity is significantly reduced and PIAS3 activity is significantly enhanced as the concentration of miR-18a decreases after using miR-18a inhibitor. Application of miR-18a inhibitor seems to rescue miR-18a induced down-regulation of PIAS3. Results of our designed HPN came out to be in agreement with experimental results. Simulations of miR-18a, pSTAT3 and PIAS3 in normal, HCC, sorafenib resistant HCC and sorafenib resistant HCC cells after the use of miR-18a inhibitor are shown in Fig. 8.

### Simulating the dynamic behaviour of miR-20a and its target MCL1 in sorafenib resistance

Study conducted by Tai *et al*., showed higher level of MCL1 expression in sorafenib resistant cell line^40^. miR-20a directly targeted MCL1 and decreased its protein level in HCC cells^45^. Approximately 4 fold increment was found in MCL1 expression of HCC cells as compared to normal and 3 fold increase was found in sorafenib resistant cells as compared to sorafenib sensitive HCC cells. Simulation results of our designed HPN model showed the respective fold increments in MCL1 level. Furthermore, simulations revealed that miR-20a mimic significantly reduced the level of MCL1 whose expression is observed to be increased in sorafenib resistant HCC cells. After calculating fold changes of respective proteins using ImageJ software, tokens were assigned to HPN model. Simulations of miR-20a and MCL1 in normal, HCC, sorafenib resistant HCC and sorafenib resistant HCC cells after the use of miR-20a mimic are shown in Fig. 9.

### Analysis of individual and combinatorial effects of miR-17-92 cluster inhibitors and mimics on sorafenib resistance

**Figure 10:**
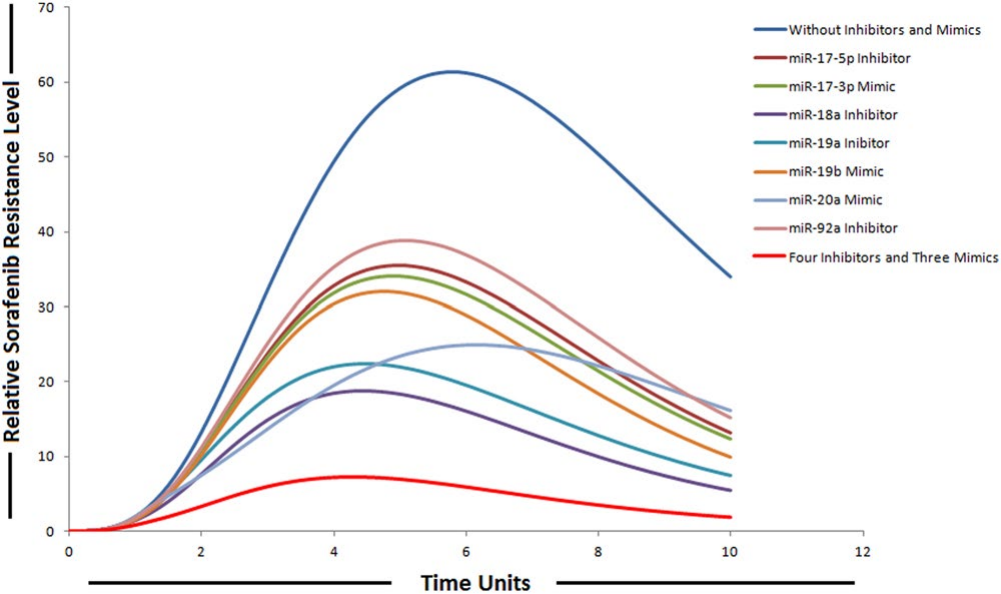
Simulation of individual and combinatorial effect of miR-17-92 cluster inhibitors and mimics on sorafenib resistance. Time units are represented on x-axis with relative sorafenib resistance level on y-axis.

The ability of miRNAs to target multiple genes within a signaling cascade engenders a novel way of thinking about modulating pathways. Instead of relying on a one-inhibitor-one-target model, multiple components in a signaling network can be modulated by a miRNA resulting in a potent impact on the respective pathway. Therefore, we explored the possibility for synergies among the members belonging to miR-17-92 cluster upon combinatorial utilization of miRNA mimics and inhibitors based on the experimental evidences provided by various studies. We then performed *in-silico* simulations to qualitatively elucidate the effect on sorafenib resistance. We found that combining four inhibitors (miR-17-5p, miR-19a, miR-92a and miR-18a) and three mimics (miR-19b, miR-20a and miR-17-3p) resulted in a significant reduction in relative sorafenib resistance levels as compared to individual effect (Fig. 10). Anti-miR-combination effects of two or more combinations were not considered. The anti-miR-17-5p specifically antagonizes the inhibition effect of miR-17-5p, which targets PTEN and E2F1; anti-miR-92a antagonizes the inhibition effect of miR-92a, which targets PTEN; anti-miR-19a antagonizes the inhibition effect of miR-19a, which targets SOCS3; anti-miR-18a antagonizes the inhibition effect of miR-18a, which targets PIAS3; miR-17-3p mimic augment the inhibition effect of miR-17-3p, which targets vimentin; miR-19b mimic augment the inhibition effect of miR-19b, which targets Rac and miR-20a mimic augment the inhibition effect of miR-20a, which targets Mcl1. Modeling miR-17-5p, miR-19a, miR-92a and miR-18a down-regulation and miR-19b, miR-20a and miR-17-3p over-expression using the experimental conditions reveals that the concentrations of miRNA-target proteins are inversely correlated with the miRNA gene expression levels. We modeled the activities of miRNA inhibitors by introducing the inhibitor arcs in the sorafenib resistant pathway. The simulation results depicted this phenomenon as clearly shown in Fig. 10. It was observed that the relative levels of sorafenib resistance were significantly reduced after the combined use of four inhibitors and three mimics belonging to miR-17-92 cluster. Based on our modeling results we suggested that the combined use might combat drug resistance in HCC, as we found significant impact on sorafenib resistance. We assumed same drug dose for each inhibitor and same for each mimic. In order to model mimics with same drug dose, instances of discrete places are represented by tokens; whereas, all proposed drugs (mimics) in our designed model have continuous source transitions. Continuous source transition is always enabled and when fired for equal runs and equal time units, can fire equal number of tokens according to the directed arc weight (by default 1 for each run). Thus, it can be assumed that after same time intervals, same number of tokens will be fired for each of the mimic in the model. Because source transitions are unconditionally firable, their average token flow rates are just their firing rates. Through transition firings, the source can influence the number of tokens assigned to the target, called the *token-count*, modeling this way enables the signals to propagate through directed protein interactions in a cellular pathway. In this way, transitions may have rates corresponding to the relative concentrations of reactants and thus may be used to model kinetics of enzymatic reactions and biological interactions. For inhibitors, we used sink transitions and inhibitor arcs. The firing of sink transitions corresponds to taking one token away. Inhibitor arcs are used to model inhibitory experiments in the signaling pathway to analyze overall systemic effects of various inhibitors. In simulations of individual miRNA inhibitor effects, we simulated the inhibition of miRNAs by setting its gene expression level to lowest by decreasing the firing rate of source transition, while all other conditions remained identical. In simulations of individual mimic effects, increments in the expression of miRNAs were obtained by increasing the firing rate of source transition of their respective genes, while all other conditions remained identical. In these simulation experiments, expression of other miRNAs was omitted to examine the effect of individual miRNA regulation. We demonstrated that collective use of inhibitors and mimics could exert a greater impact on the signaling pathway and might significantly revert sorafenib resistant state to sorafenib sensitive state.

Moreover, we also focused on the dynamic behaviour of SOCS3-STAT3 negative feedback loop being regulated by miR-19a and miR-18a in our proposed sorafenib resistant HPN model. Network motifs including feedback and feed forward loops play an imperative role in determining the dynamic behaviour of biological signaling networks and can generate a wide variety of behaviors, especially when they are interconnected^58–60^. Both positive and negative feedback types can lead to network instabilities. Several previous studies have reported the presence of regulatory feedback loops between miRNAs and the STAT3 pathway in different cancer contexts including IL-6-STAT3-miR-24/miR-629-HNF4-miR-124^61^ and IL-6RSTAT3-NF-B-Lin-28-let-7a^62^. In our proposed miR-17-92 mediated sorafenib resistant HPN model, we focused on SOCS3-STAT3 negative feedback loop being regulated by miR-19a and miR-18a.

Both the output node and the input node (i.e. the node whose activation is augmented by a downstream node) in negative feedback loop seems to be more effective targets than other nodes in the network. Here, we uncovered that miR-19a promotes sorafenib resistance through repression of SOCS3 and upregulation of STAT3 in STAT3-SOCS3 negative feedback loop. The defining feature of a negative feedback motif is an activator–inhibitor pair in which the activator (STAT3 in this case) induces expression or activity of the inhibitor (SOCS3). But this activator-inhibitor paradigm cannot completely describe the molecular circuits within the cell as they may need to contend with physical realities within the cell such as sub-cellular compartmentalization, the biochemistry of molecular interactions or protein half-life. Thus, accurate functioning of a negative feedback circuit may rely upon biochemical properties other than the activator-responsive control of the inhibitor^63^.

### MiR-19a promotes sorafenib resistance by augmenting STAT3 and repressing SOCS3 in STAT3-SOCS3 negative feedback loop

SOCS3 regulates STAT3 activation through a negative feedback loop. SOCS3 is activated by STAT3 and functions by inhibiting JAK2 leading to decreased STAT3. Studies have reported prolonged activation of STAT3 after conditional knock out of SOCS3 in murine liver and macrophages^64^. Under physiological conditions in normal cells, the activation of STAT proteins is rapid and transient because of negative regulation by proteins such as SOCS3 and PIAS3 as illustrated in simulations (Figure S2). Multiple negative regulators, such as SOCS3 and PIAS3, have fundamental roles to keep STAT3 activity at low levels in normal conditions. In our proposed miR-17-92 mediated sorafenib resistant signaling cascade, STAT3 pathway is under the control of two miRNAs including miR-19a and miR-18a. Clinically, the upregulation of STAT3 in cancer cells is also linked with severe drug resistance and worst prognosis. Furthermore SOCS3 is known to play an important role in liver carcinogenesis and were observed to be down-regulated in carcinogenesis. Previously SOCS3 has also been observed to play an important role in mediating cisplatin resistance^65^. In addition, miR-18a is known to inhibit PIAS3 which is another negative regulator of STAT3. Based on previous experimental findings and evidences, we hypothesized that perturbation of this network through miR-19a inhibitor and miR-18a inhibitor might help to combat sorafenib resistance by downregulating STAT3. To further unravel the mechanism by which inhibition of miR-19a and miR-18a can disturb this negative feedback loop and induces sorafenib resistance, we performed simulations in the presence and absence of miR-19a/miR-18a inhibitor (Figure S2).

We speculated that increased expression of miR-19a and miR-18a might be responsible for the upregulation of STAT3 activity in sorafenib resistant HCC cells. As activation of STAT3 is influenced by a variety of intrinsic pathway components, therefore understanding how this signaling is controlled presents a huge challenge which may be best addressed by computational modeling. Our simulation results revealed that sorafenib resistant pathway is more sensitive to miR-19a inhibition than miR-18a inhibition. The study conducted by Mahdavi *et al*., reported that SOCS3 is the most sensitive parameter for manipulating STAT3 pathway output^66^ which is further supporting our simulation results as SOCS3 is the target of miR-19a. Furthermore, our simulations revealed that if both miR-19a and miR-18a inhibitors are used simultaneously, they will dominate the response by significantly reducing STAT3 (Figure S2).

**Figure 11:**
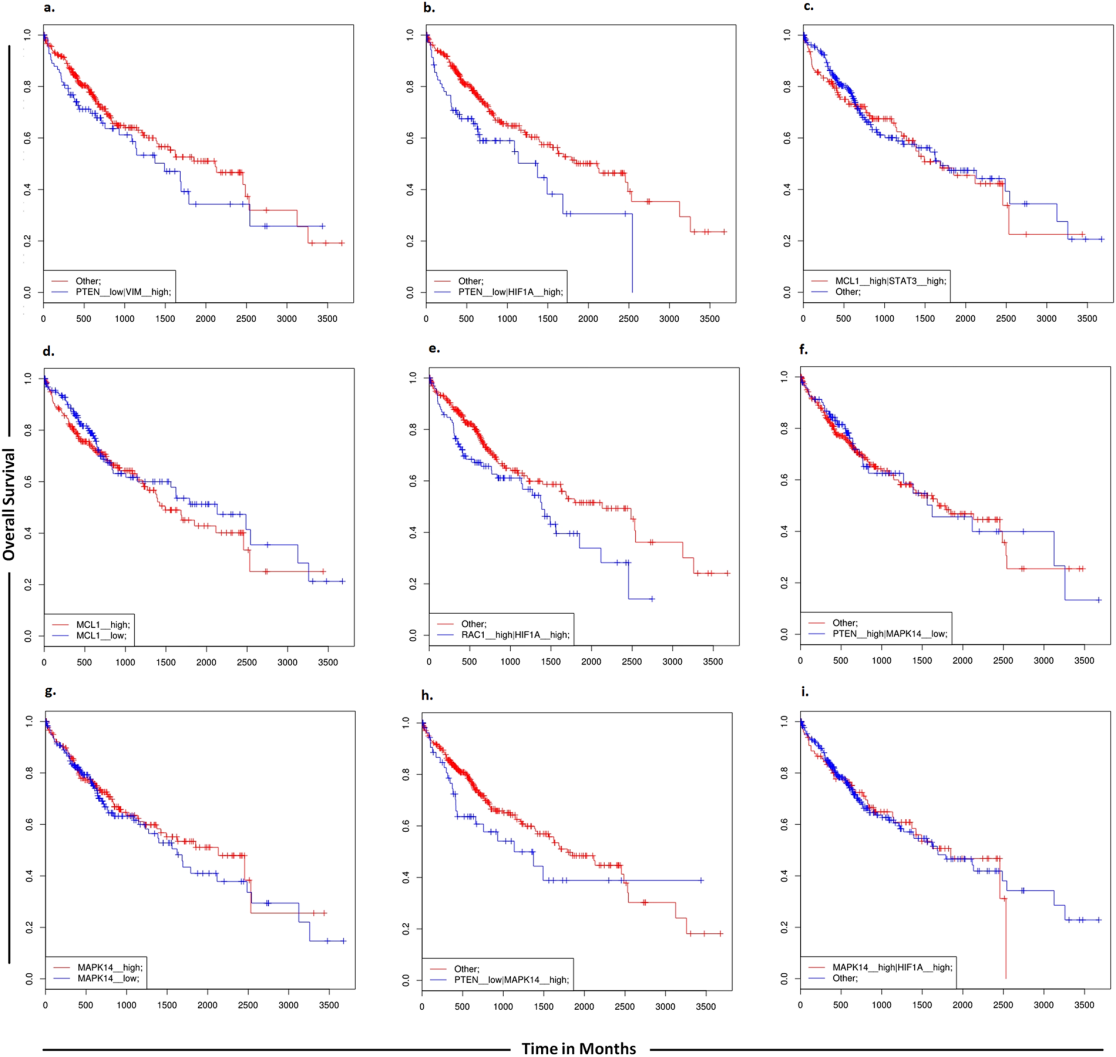
SynTarget survival analyses. SynTarget was used to assess the synergistic survival relationships resulting from expression of combinations of genes targeted by miR-17-92 cluster.

Furthermore, in order to determine a correlation between targets of miR-17-92 cluster and overall survival of HCC patients, we carried out the analysis using the SynTarget software. Low PTEN and high vimentin expressions were found to be linked with poor survival outcome. Low level of PTEN and high level of expression of HIF-1Α were also found to be linked with poor survival outcome. When the expression of either MCL1 and STAT3 genes or MCL1 alone was high, survival outcomes of patients were decreased. High level of PTEN and low level of MAPK14 were found to be associated with increased overall survival of HCC patients. Similarly high level of either MAPK14 and HIF-1α genes or MAPK14 alone was found to be linked with poor survival of HCC patients (Fig. 11).

**Figure 12:**
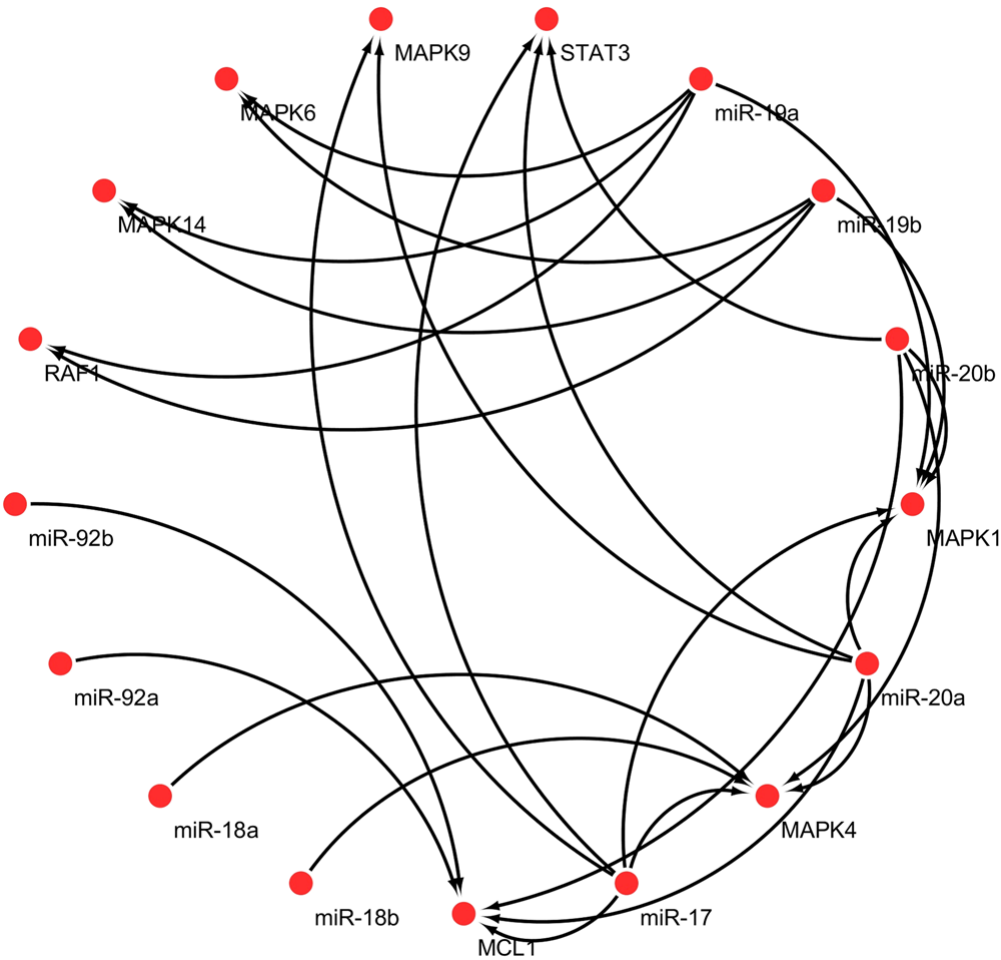
miRNA pharmacogenomic set (members belonging to miR-17-92 cluster, their target genes and sorafenib) as predicted by Pharmaco-miR.

Using default search parameters of Pharmaco-miR, 27 sets containing members of miR-17-92 cluster and sorafenib were identified (Table 5 and Fig. 12). Interplay of miR-17-92 modulators have been shown in the designed models and thus their overall effect on sorafenib resistance has been revealed. Simulation results of the integrated network along with inhibitors and mimics of miR-17-92 cluster revealed significant reduction in sorafenib resistance. Results of the current findings were consistent with experimental observations.

**Table 5:** Pharmacogenomic set of members belonging to miR-17-92 cluster, their targets and sorafenib as predicted by Pharmaco-miR.

Resistance drug	Deregulated miRNA	Pharmaco-miR putative effector gene
Sorafenib	miR-20b	STAT3
Sorafenib	miR-20a	STAT3
Sorafenib	miR-17	STAT3
Sorafenib	miR-20b	MCL1
Sorafenib	miR-20a	MCL1
Sorafenib	miR-17	MCL1
Sorafenib	miR-92a	MCL1
Sorafenib	miR-92b	MCL1
Sorafenib	miR-19a	RAF1
Sorafenib	miR-19b	RAF1
Sorafenib	miR-19a	MAPK1
Sorafenib	miR-19b	MAPK1
Sorafenib	miR-20b	MAPK1
Sorafenib	miR-20a	MAPK1
Sorafenib	miR-17	MAPK1
Sorafenib	miR-19a	MAPK14
Sorafenib	miR-19b	MAPK14
Sorafenib	miR-20a	MAPK9
Sorafenib	miR-17	MAPK9
Sorafenib	miR-20b	MAPK4
Sorafenib	miR-20a	MAPK4
Sorafenib	miR-17	MAPK4
Sorafenib	miR-18b	MAPK4
Sorafenib	miR-18a	MAPK4
Sorafenib	miR-19a	MAPK6
Sorafenib	miR-19b	MAPK6

Cancer imposes a considerable economic burden on societies and is projected to increase significantly over the next two to three decades. Today’s data sets are of such magnitude and complexity that advanced bioinformatics methods are essential to their integration, management and dissemination. Drug resistance in cancer represents an insidious barrier which demands further research and treatment development. The main aim of our study was to understand drug resistance in HCC and to discover multiple targets that may overcome this problem. This is a difficult obstacle to overcome. Here, a HPN based systems biological model of miRNA/EGFR/IL-6 signaling cascade was constructed. Understanding underlying molecular and cellular mechanisms of ncRNAs mediated drug resistance might have a great clinical potential for the development of rational therapeutic combinations. We are hoping our research leads to the system that can be used for therapeutic decision in sorafenib resistant HCC patients.

Various studies performed on drug-resistant cells have reported deregulated miRNA profile, but target genes of these miRNAs remain to be identified. The scarcity of such studies highlights the need for further research to interrelate miRNA, genes and drugs. Expression of the EGFR/IL-6 pathway members PI3K, ERK, AKT, STAT3, JAK-2, MCL1, P38MAPK, E2F1 and WIP1 have previously shown to be crucial for sorafenib resistance. All these crucial factors are target of members belonging to miR-17-92 cluster. Current study aims to determine synergistic effect of miR-17-92 cluster inhibitors/mimics on sorafenib resistant HCC cells. Despite the outstanding obstacles, targeting of miRNA function by miRNA-based drugs either as mimics or inhibitors has become a practical option for the modulation of many aspects of human pathologies.

Study conducted by Vaira *et al*., revealed elevated level of miR-17-92 cluster members in HCC tissues which resisted the anticancer efficacy of sorafenib suggesting that they could limit the anticancer efficacy of sorafenib in HCC patients and miRNA levels could restore sensitivity to sorafenib. Their study was based on validating elevated miR-425-3p levels in patients, who respond better to sorafenib therapy. They found a correlation between higher miR-425-3p level, apoptosis and reduced cell motility in sorafenib treated HCC cell lines further authenticating these findings. In patients with low miR-425-3p level, the authors observed high level of miR-17-92 cluster members although they did not validate this but it is intriguing why patients who resisted anticancer efficacy of sorafenib have increased expression of members belonging to miR-17-92 cluster^67^.

The hypothetical implication of the critical role of the miR-17-92/EGFR/IL-6 axis in HCC will be a significant contribution that leads to an in-depth understanding of sorafenib resistance. We firmly believe that sorafenib may be used more efficiently in combination with miR-17-92 targeted therapy to make a more specific treatment. Future research should focus on targeting sorafenib resistant HCC cells using inhibitors and mimics of miR-17-92 cluster that could be highly efficient therapeutic strategy acting downstream of the EGFR/IL-6 axis in signaling cascade. New mechanisms predicted by our HPN model suggest that combined use of four inhibitors (miR-17-5p, miR-19a, miR-92 and miR-18a) and three mimics (miR-17-3p, miR-19b and miR-20a) of miR-17-92 cluster may overcome sorafenib resistance in HCC via regulating the related pathways including EGFR and IL-6.

Our approach is the first step towards addressing the testable hypothesis that the behavior of miRNAs belonging to miR-17-92 cluster might potentially contributes to the sorafenib resistant phenotype in HCC. Through simulations that capture the intricate relations between miRNAs and their targets, this model explores an innovative perspective about the distinctive yet integrated roles of miRNAs in sorafenib resistance, and it will help to elucidate the dysregulated miRNA profiles found in resistant cancer cells. Furthermore, our HPN model can be easily extended by replacing certain components and parameters in the model and can also serve as basis of theoretical knockout experiments. They can be constructed by deleting the corresponding place and adjacent transitions and nominate possible indicators for biological exploration.

## Methods

### Petri Nets

PN, a directed, finite, bipartite labeled graph is mathematical modeling language introduced by Carl Adam Petri in 1962 which is becoming the reference modeling formalism for regulatory networks^68^. PN is comprised of places, transitions, arcs and tokens. A place signifies possible state of the system and can hold tokens (zero or more) as its content. Transitions are actions or events which cause the change of state and arcs connect a place (input place) to transition or a transition to place (output). A transition with these arcs defines a firing rule and its execution usually depends on the availability of a specific number of tokens in the places. Graphically, places are illustrated as circles, transitions as squares and tokens are shown as dots or integers in the places.

Several enhanced PNs have been used to model biological phenomena which can help to get better level of understanding. HPN is an extension of PN, since it includes both continuous and discrete places/transitions, it can easily handle biological and biochemical factors. As protein concentration dynamics behaves continuously together with discrete switches; therefore, biological pathways can be considered as hybrid systems. HPNs present a high level of integration and have been recognized as powerful modeling tools for efficient modeling and analysis of biological pathways^69^. They are known to have a strong mathematical semantics, and this formal basis makes it possible to make strong statements about the characteristics of the processes being modeled. Because of its formal approach, processes are forced to be defined in a precise manner. This in turn prevents the occurrence of uncertainties, ambiguities and contradictions. This formal basis combined with the nice graphical representation makes it possible to argue about processes, and thereby enables the possible establishment of certain patterns^70^. Moreover, they can represent system behavior even when the biological mechanism is not fully understood, by combining different levels of abstraction in a single model and enable users to verify system properties, verify system soundness, and simulate dynamic behavior. Furthermore, previous studies have also shown strong prediction power of PNs by experimentally validating their models^71, 72^.

### Construction of the PN

The steps implicated in HPN model generation were; literature survey to extract the most significant miR-17-92 cluster determinants of sorafenib resistance; abstraction of the extracted pathway; generation of HPN model; scrutinization and verification of the model. In order to model our pathway, Snoopy-a unified PN tool^73^, comprising of a family of PN classes is employed. In the designed HPN model, places signifies gene, mRNA, miRNA, protein and compounds etc involved in sorafenib resistance and transitions depict the interactions, processes or reactions (e.g., de-/activation, de-/phosphorylation, transcription, gene silencing, chemical reactions, complex formation, transport processes etc.). Inhibitory arcs demonstrate inhibitory effects of miRNAs and signaling proteins on certain cellular processes or protein. Transitions are enabled by the number of tokens present in input/pre places. Transitions without input/pre places are called source transitions. Transitions without output/post places are called sink transitions. Here, the activated proteins/enzymes involved in the signaling pathways are represented through input transitions, whereas output transitions depict dissociation/decay. Each place contains certain amount of tokens representing the concentration of a model component. All native signaling molecules have been given arbitrary token numbers.

### Modeling miRNA-EGFR/IL-6-Specific Signaling Network

**Figure 13:**
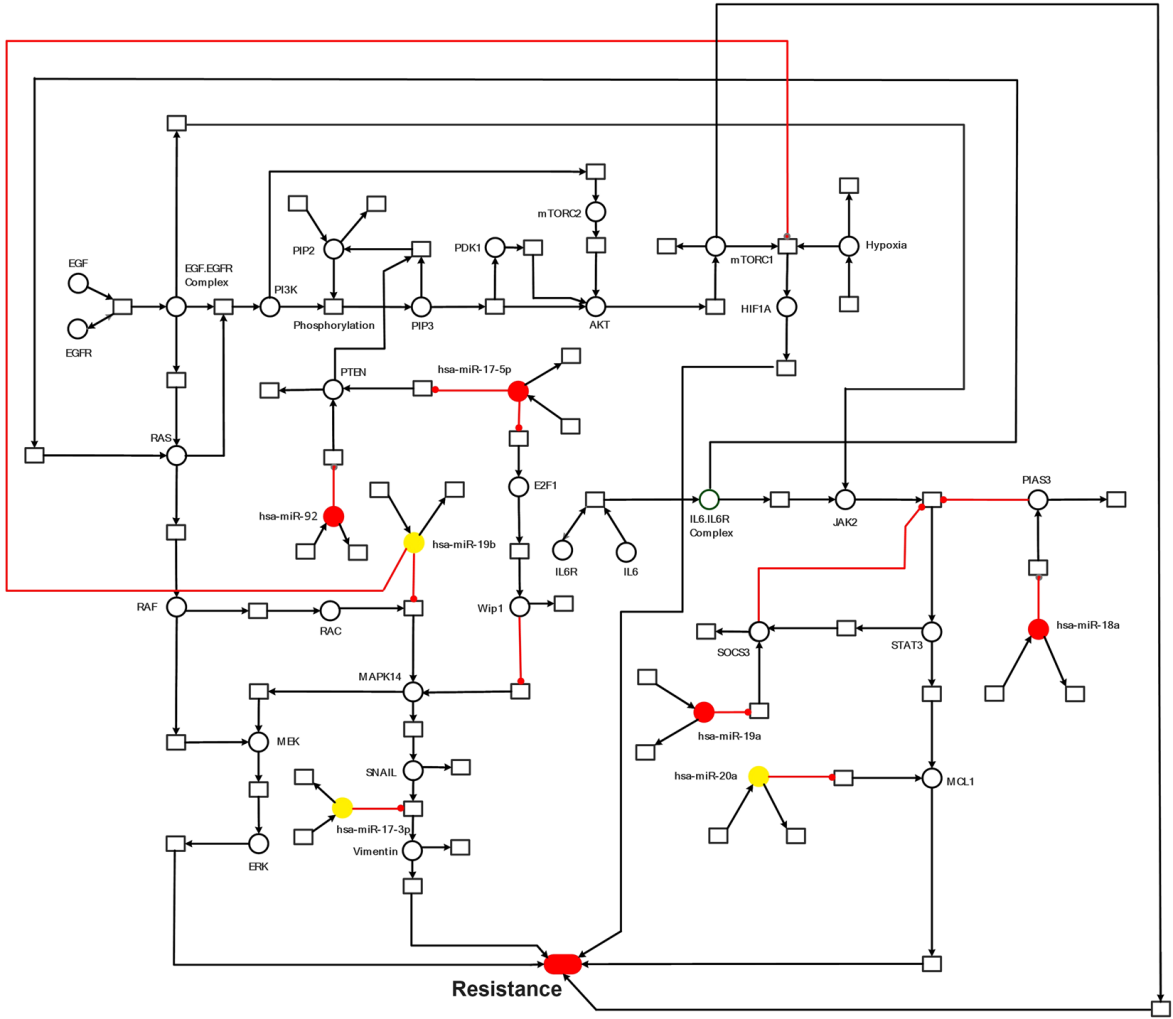
Computational modeling framework. HPN model illustrating miR-17-92 cluster mediated sorafenib resistance pathway. A standard place is demonstrated as circlel ○ representing enzymes, signaling proteins and complexes. Continuous transition is showed as square  & representing all cellular processes i.e. inhibition and activation. miRNAs with red circle  represent positive effect on sorafenib resistance while miRNAs with yellow circle  represents negative effect on sorafenib resistance.

The miR-17-92/EGFR/IL-6 signaling model was constructed using Snoopy as an HPN model. An outline of the modeled signaling network is represented in Fig. 1. In order to explore the dynamics of members belonging to miR-17-92 cluster leading to sorafenib resistant phenotype, we incorporated numerous well-characterized pathways that have been reported to play a role in sorafenib resistance. A HPN illustration of the integrated network is shown in Fig. 13. Our model includes IL-6/EGFR Signaling molecules being targeted by hsa-miR-17-5p, hsa-miR-17-3p, hsa-miR-19a, hsa-miR-19b, hsa-miR-20a, hsa-miR-18a and hsa-miR-92. We explored the contributions of individual pathways to the differential regulation of miRNAs to identify crucial and sufficient regulatory relationships during sorafenib resistance. ImageJ was used to calculate relative signal intensities of various signaling proteins. The relative intensity of detected bands was normalized.

### Setup for perturbation simulations

In order to initiate markings for the HPN model, experimentally derived fold changes of EGFR/IL-6 signaling proteins were compared in normal vs HCC cells and sorafenib resistant vs sorafenib sensitive HCC cells. The experimental states for normal vs HCC and sorafenib resistant vs HCC were derived from western blot results produced from various studies and analyzed by ImageJ. The number of tokens allocated to entity was directly proportional to the intensity of the band on the western blot.

### Simulations of preliminary signaling model

Firstly, parameter fitting of the preliminary model with the wild type data was carried out. Several parameters were examined so that the simulations could match the experimental profiles. Simulations were able to successfully match experimental profiles. We examined western blot images reported in various previous studies. We used gel analysis function of ImageJ software and calculated the total intensity of the band. The intensities of alterations were expressed as fold changes with respect to their nominal values.

### Identification of circular-RNAs (circRNAs) acting as sponges for members belonging to miR-17-92 cluster

Number of circRNAs have been identified, and some of them have been revealed to be replete with miRNA binding sites, capable of binding multiple miRNAs and prevent them from interacting with targets mRNAs^74^. These studies imply a role for circRNAs in post-transcriptional regulation. We analyzed various circular RNA databases including CircBase (http://circbase.org/)^75^, circ2Traits (http://gyanxet-beta.com/circdb/)^76^, CircNet (http://circnet.mbc.nctu.edu.tw/)^77^, CircInteractome (http://circinteractome.nia.nih.gov/)^78^ as well as various studies conducted on circRNAs to find out circRNAs linked to HCC and acting as sponge to members belonging to miR-17-92 cluster. 127 circular RNAs were found to have binding sites for miR-17-92 cluster members.

### Survival and miRNA pharmacogenomics analysis

Identifying target combinations with synergistic effects on cancer remains a challenging task and is attracting significant interests, particularly for the development of combined anticancer therapies. Prognosis is the likely outlook (person’s life expectancy and cure rate) of a disease. In order to find out the effect of various targets of members belonging to miR-7-92 cluster on overall survival of patients with HCC, SynTarget^79^ was used. We utilized this tool to assess the synergistic relationships of critical targets of miR-17-92 involved in sorafenib resistance. SynTarget contain large-scale gene expression data sets comprising of eight diverse cancers with the possibility to choose clinically important subtypes.

Furthermore, a drug-miRNA expression database, Pharmaco-miR was utilized to find out miRNA-gene-drug correlation^18^. This database derives information from experimental data as well as computational predictions to correlate miRNA expression, its target gene and a drug related with the gene.

### Data availability

All data generated or analysed during this study are included in the article (and its Supplementary Information file).

## Electronic supplementary material

Supplementary Information

## References

[1] DeVita VT, Chu E (2008). A history of cancer chemotherapy. Cancer research.

[2] Garraway LA, Janne PA (2012). Circumventing cancer drug resistance in the era of personalized medicine. Cancer Discov.

[3] Zheng T, Wang J, Chen X, Liu L (2010). Role of microRNA in anticancer drug resistance. Int J Cancer.

[4] Feizabadi MS, Witten TM (2015). Modeling drug resistance in a conjoint normal-tumor setting. Theor Biol Med Model.

[5] Magee, P., Shi, L. & Garofalo, M. Role of microRNAs in chemoresistance. *Annals of translational medicine* **3** (2015). 10.3978/j.issn.2305-5839.2015.11.32 PMC4690999 26734642

[6] Garofalo M, Croce CM (2013). MicroRNAs as therapeutic targets in chemoresistance. Drug Resistance Updates.

[7] Neesse A, Gress TM (2015). Emerging role of microRNAs to tackle drug resistance in pancreatic cancer. Gut.

[8] Wen, L. *et al*. Regulation of Multi-drug Resistance in hepatocellular carcinoma cells is TRPC6/Calcium Dependent. *Scientific reports* **6**, doi:10.1038/srep23269 (2016). 10.1038/srep23269 PMC4806320 27011063

[9] Xiu P, Dong X-F, Li X-P, Li J (2015). Clusterin: Review of research progress and looking ahead to direction in hepatocellular carcinoma. World J Gastroenterol.

[10] Park JW (2015). Global patterns of hepatocellular carcinoma management from diagnosis to death: the BRIDGE Study. Liver International.

[11] Ingle PV (2016). Development and novel therapeutics in hepatocellular carcinoma: a review. Therapeutics and clinical risk management.

[12] Shao YY, Hsu CH, Cheng AL (2015). Predictive biomarkers of sorafenib efficacy in advanced hepatocellular carcinoma: Are we getting there?. World J Gastroenterol.

[13] Xia F (2016). Adjuvant sorafenib after heptectomy for Barcelona Clinic Liver Cancer-stage C hepatocellular carcinoma patients. World J Gastroenterol.

[14] Llovet JM (2008). Sorafenib in advanced hepatocellular carcinoma. N Engl J Med.

[15] Zhai B, Sun XY (2013). Mechanisms of resistance to sorafenib and the corresponding strategies in hepatocellular carcinoma. World J Hepatol.

[16] Xu Y (2016). MicroRNA-122 confers sorafenib resistance to hepatocellular carcinoma cells by targeting IGF-1R to regulate RAS/RAF/ERK signaling pathways. Cancer letters.

[17] Berasain C (2013). Hepatocellular carcinoma and sorafenib: too many resistance mechanisms?. Gut.

[18] Rukov JL, Wilentzik R, Jaffe I, Vinther J, Shomron N (2013). Pharmaco-miR: linking microRNAs and drug effects. Brief Bioinform.

[19] Bonauer A, Dimmeler S (2009). The microRNA-17-92 cluster: still a miRacle?. Cell Cycle.

[20] Xiang J, Wu J (2010). Feud or Friend? The Role of the miR-17-92 Cluster in Tumorigenesis. Curr Genomics.

[21] Cioffi M (2015). The miR-17-92 cluster counteracts quiescence and chemoresistance in a distinct subpopulation of pancreatic cancer stem cells. Gut.

[22] Zhu H, Yang SY, Wang J, Wang L, Han SY (2016). Evidence for miR-17-92 and miR-134 gene cluster regulation of ovarian cancer drug resistance. Eur Rev Med Pharmacol Sci.

[23] Zhou P (2016). miR-17-92 plays an oncogenic role and conveys chemo-resistance to cisplatin in human prostate cancer cells. International journal of oncology.

[24] Miles, R. R., Rodic, V., Barth, M. J., Cairo, M. S. & Hermiston, M. L. Microrna (miR)-17-92 Contributes to Therapy Resistance in Burkitt Lymphoma Cells. *Blood* **128** (2016).

[25] Blair RH, Trichler DL, Gaille DP (2012). Mathematical and statistical modeling in cancer systems biology. Advances in Systems Immunology and Cancer.

[26] Machado D (2011). Modeling formalisms in systems biology. AMB express.

[27] Materi W, Wishart DS (2007). Computational systems biology in drug discovery and development: methods and applications. Drug discovery today.

[28] Ruths D, Muller M, Tseng JT, Nakhleh L, Ram PT (2008). The signaling petri net-based simulator: a non-parametric strategy for characterizing the dynamics of cell-specific signaling networks. PLoS Comput Biol.

[29] Chaouiya C (2007). Petri net modelling of biological networks. Brief Bioinform.

[30] Chen KF (2011). Activation of phosphatidylinositol 3-kinase/Akt signaling pathway mediates acquired resistance to sorafenib in hepatocellular carcinoma cells. J Pharmacol Exp Ther.

[31] Shan SW (2013). Mature miR-17-5p and passenger miR-17-3p induce hepatocellular carcinoma by targeting PTEN, GalNT7 and vimentin in different signal pathways. J Cell Sci.

[32] Yang F (2010). miR‐17‐5p Promotes migration of human hepatocellular carcinoma cells through the p38 mitogen‐activated protein kinase‐heat shock protein 27 pathway. Hepatology.

[33] Hershko T, Korotayev K, Polager S, Ginsberg D (2006). E2F1 modulates p38 MAPK phosphorylation via transcriptional regulation of ASK1 and Wip1. J Biol Chem.

[34] Rudalska R (2014). *In vivo* RNAi screening identifies a mechanism of sorafenib resistance in liver cancer. Nat Med.

[35] van Malenstein H (2013). Long-term exposure to sorafenib of liver cancer cells induces resistance with epithelial-to-mesenchymal transition, increased invasion and risk of rebound growth. Cancer Lett.

[36] Zhao B (2013). Expression and significance of PTEN and miR-92 in hepatocellular carcinoma. Mol Med Rep.

[37] Hung CL, Yen CS, Tsai HW, Su YC, Yen CJ (2015). Upregulation of MicroRNA-19b predicts good prognosis in patients with hepatocellular carcinoma presenting with vascular invasion or multifocal disease. BMC Cancer.

[38] Liu Y, Li PK, Li C, Lin J (2010). Inhibition of STAT3 signaling blocks the anti-apoptotic activity of IL-6 in human liver cancer cells. J Biol Chem.

[39] Ezzoukhry Z (2012). EGFR activation is a potential determinant of primary resistance of hepatocellular carcinoma cells to sorafenib. Int J Cancer.

[40] Tai WT (2012). Dovitinib induces apoptosis and overcomes sorafenib resistance in hepatocellular carcinoma through SHP-1-mediated inhibition of STAT3. Mol Cancer Ther.

[41] Tamiya T, Kashiwagi I, Takahashi R, Yasukawa H, Yoshimura A (2011). Suppressors of cytokine signaling (SOCS) proteins and JAK/STAT pathways. Arteriosclerosis, thrombosis, and vascular biology.

[42] Croker BA (2003). SOCS3 negatively regulates IL-6 signaling *in vivo*. Nat Immunol.

[43] Collins AS, McCoy CE, Lloyd AT, O’Farrelly C, Stevenson NJ (2013). miR-19a: an effective regulator of SOCS3 and enhancer of JAK-STAT signalling. PLoS One.

[44] Brock M (2011). MicroRNA-18a enhances the interleukin-6-mediated production of the acute-phase proteins fibrinogen and haptoglobin in human hepatocytes. J Biol Chem.

[45] Fan MQ (2013). Decrease expression of microRNA-20a promotes cancer cell proliferation and predicts poor survival of hepatocellular carcinoma. J Exp Clin Cancer Res.

[46] Hashiba T (2015). Regorafenib inhibits Erk signaling and suppresses the growth of sorafenib-resistant cells in human hepatocellular carcinoma. Hepatology.

[47] Dong-Dong L, Xi-Ran Z, Xiang-Rong C (2003). Expression and significance of new tumor suppressor gene PTEN in primary liver cancer. J Cell Mol Med.

[48] Yang Z, Yi J, Li X, Long W (2005). Correlation between loss of PTEN expression and PKB/AKT phosphorylation in hepatocellular carcinoma. J Huazhong Univ Sci Technolog Med Sci.

[49] Wang H (2014). Activation of phosphatidylinositol 3-kinase/Akt signaling mediates sorafenib-induced invasion and metastasis in hepatocellular carcinoma. Oncology reports.

[50] Hu TH (2003). Expression and prognostic role of tumor suppressor gene PTEN/MMAC1/TEP1 in hepatocellular carcinoma. Cancer.

[51] Ruan ZP (2012). PTEN enhances the sensitivity of human hepatocellular carcinoma cells to sorafenib. Oncol Res.

[52] Yang F (2010). miR-17-5p Promotes migration of human hepatocellular carcinoma cells through the p38 mitogen-activated protein kinase-heat shock protein 27 pathway. Hepatology.

[53] Zheng J, Dong P, Gao S, Wang N, Yu F (2013). High expression of serum miR-17-5p associated with poor prognosis in patients with hepatocellular carcinoma. Hepatogastroenterology.

[54] Yin Y, Shen W (2008). PTEN: a new guardian of the genome. Oncogene.

[55] Hu L (2004). Association of Vimentin overexpression and hepatocellular carcinoma metastasis. Oncogene.

[56] Huang JF (2013). Hepatitis B virus X protein (HBx)-related long noncoding RNA (lncRNA) down-regulated expression by HBx (Dreh) inhibits hepatocellular carcinoma metastasis by targeting the intermediate filament protein vimentin. Hepatology.

[57] Liang Y (2013). Hypoxia‐mediated sorafenib resistance can be overcome by EF24 through Von Hippel‐Lindau tumor suppressor‐dependent HIF‐1α inhibition in hepatocellular carcinoma. Hepatology.

[58] Kolch W, Halasz M, Granovskaya M, Kholodenko BN (2015). The dynamic control of signal transduction networks in cancer cells. Nature Reviews Cancer.

[59] Cloutier M, Wang E (2011). Dynamic modeling and analysis of cancer cellular network motifs. Integrative Biology.

[60] Tian X-J, Zhang X-P, Liu F, Wang W (2009). Interlinking positive and negative feedback loops creates a tunable motif in gene regulatory networks. Physical Review E.

[61] Hatziapostolou M (2011). An HNF4α-miRNA inflammatory feedback circuit regulates hepatocellular oncogenesis. Cell.

[62] Iliopoulos D, Hirsch HA, Struhl K (2009). An epigenetic switch involving NF-κB, Lin28, Let-7 MicroRNA, and IL6 links inflammation to cell transformation. Cell.

[63] Fagerlund R (2015). Anatomy of a negative feedback loop: the case of IκBα. Journal of The Royal Society Interface.

[64] Croker BA (2003). SOCS3 negatively regulates IL-6 signaling *in vivo*. Nature immunology.

[65] Ru P, Steele R, Hsueh EC, Ray RB (2011). Anti-miR-203 upregulates SOCS3 expression in breast cancer cells and enhances cisplatin chemosensitivity. Genes & cancer.

[66] Mahdavi A, Davey RE, Bhola P, Yin T, Zandstra PW (2007). Sensitivity analysis of intracellular signaling pathway kinetics predicts targets for stem cell fate control. PLoS Comput Biol.

[67] Vaira, V. *et al*. MicroRNA-425-3p predicts response to sorafenib therapy in patients with hepatocellular carcinoma. *Liver Int*, doi:10.1111/liv.12636 (2014). 10.1111/liv.12636 25040368

[68] Pinney JW, Westhead DR, McConkey GA (2003). Petri Net representations in systems biology. BIOCHEM SOC T.

[69] Alla, H. & Ghomri, L. In *Simulation Conference (WSC)*, *Proceedings of the* 2012 *Winter*. 1–8 (IEEE).

[70] Bos, W. Modeling biological systems using Petri nets. Department of Electrical Engineering, Mathematics and Computer Science, University of Twente, Netherland (2008).

[71] Jacobsen A (2016). Construction and Experimental Validation of a Petri net Model of Wnt/β-catenin Signaling. PLoS One.

[72] Polak ME, Ung CY, Masapust J, Freeman TC, Ardern-Jones MR (2017). Petri Net computational modelling of Langerhans cell Interferon Regulatory Factor Network predicts their role in T cell activation. Scientific reports.

[73] Heiner, M., Herajy, M., Liu, F., Rohr, C. & Schwarick, M. Snoopy – A Unifying Petri Net Tool. in *Application and Theory of Petri Nets*, 398–407 (2012).

[74] Thomas LF, Sætrom P (2014). Circular RNAs are depleted of polymorphisms at microRNA binding sites. Bioinformatics.

[75] Glazar P, Papavasileiou P, Rajewsky N (2014). circBase: a database for circular RNAs. RNA.

[76] Ghosal S, Das S, Sen R, Basak P, Chakrabarti J (2013). Circ2Traits: a comprehensive database for circular RNA potentially associated with disease and traits. Front Genet.

[77] Liu YC (2015). CircNet: a database of circular RNAs derived from transcriptome sequencing data. Nucleic Acids Res.

[78] Dudekula DB (2015). CircInteractome: a web tool for exploring circular RNAs and their interacting proteins and microRNAs. RNA Biol.

[79] Amelio I (2016). SynTarget: an online tool to test the synergetic effect of genes on survival outcome in cancer. Cell Death & Differentiation.

[80] Wang L (2013). MicroRNA-302b suppresses cell proliferation by targeting EGFR in human hepatocellular carcinoma SMMC-7721 cells. BMC Cancer.

[81] Ling B, Chen L, Liu Q, Yang J (2014). Gene expression correlation for cancer diagnosis: a pilot study. Biomed Res Int.

[82] Ji’an, Z. *et al*. Expression of JAK2/STAT3 signaling pathway in human hepatocellular carcinoma and its significance. *Chinese Journal of General Surgery* (2016).

[83] Fleischer B (2006). Mcl-1 is an anti-apoptotic factor for human hepatocellular carcinoma. Int J Oncol.

