# Supplementary material for: MicroRNA pharmacogenomics based integrated model of miR-17-92 cluster in sorafenib resistant HCC cells reveals a strategy to forestall drug resistance
Source: Sci Rep. 2017 Sep 13;7:11448. doi: 10.1038/s41598-017-11943-1 (PMC5597599; doi:10.1038/s41598-017-11943-1)
Supplement: Supplementary file 1 — Supplementary Information [file 41598_2017_11943_MOESM1_ESM.pdf]

Supplementary Information

Title

MicroRNA pharmacogenomics based integrated model of miR-17-92 cluster in sorafenib resistant HCC cells reveals a strategy to forestall drug resistance

Authors

Faryal Mehwish Awan[1], Anam Naz[1], Ayesha Obaid[1], Aqsa Ikram[1], Amjad Ali[1], Jamil Ahmad[2], Abdul Khaliq Naveed[3], Hussnain Ahmed Janjua*[, 1]

Affiliations

[1]Atta-ur-Rahman School of Applied Biosciences (ASAB), National University of Sciences and Technology (NUST), H-12 Islamabad, Pakistan.

[2]Research Center for Modeling and Simulation (RCMS), National University of Sciences and Technology (NUST), H-12 Islamabad, Pakistan.

[3]Islamic International Medical College (IIMC), Riphah International University, Rawalpindi, Pakistan

[*]Corresponding author

Hussnain Ahmed Janjua

E-mail: janjua.hussnain@gmail.com ; hussnain.janjua@asab.nust.edu.pk

Telephone number: +92 323 5680231

Author's contact information

Faryal Mehwish Awan faryal_mehwish@yahoo.com

Anam Naz anam.naz88@live.com

Ayesha Obaid ayesha_obaid_nust@live.com

Aqsa Ikram aqsa_ikram@yahoo.com

Amjad Ali amjaduni@gmail.com

Jamil Ahmad dr.ahmad.jamil@gmail.com

Abdul Khaliq Naveed khaliqnaveed2001@yahoo.com

Hussnain Ahmed Janjua* janjua.hussnain@gmail.com ; hussnain.janjua@asab.nust.edu.pk

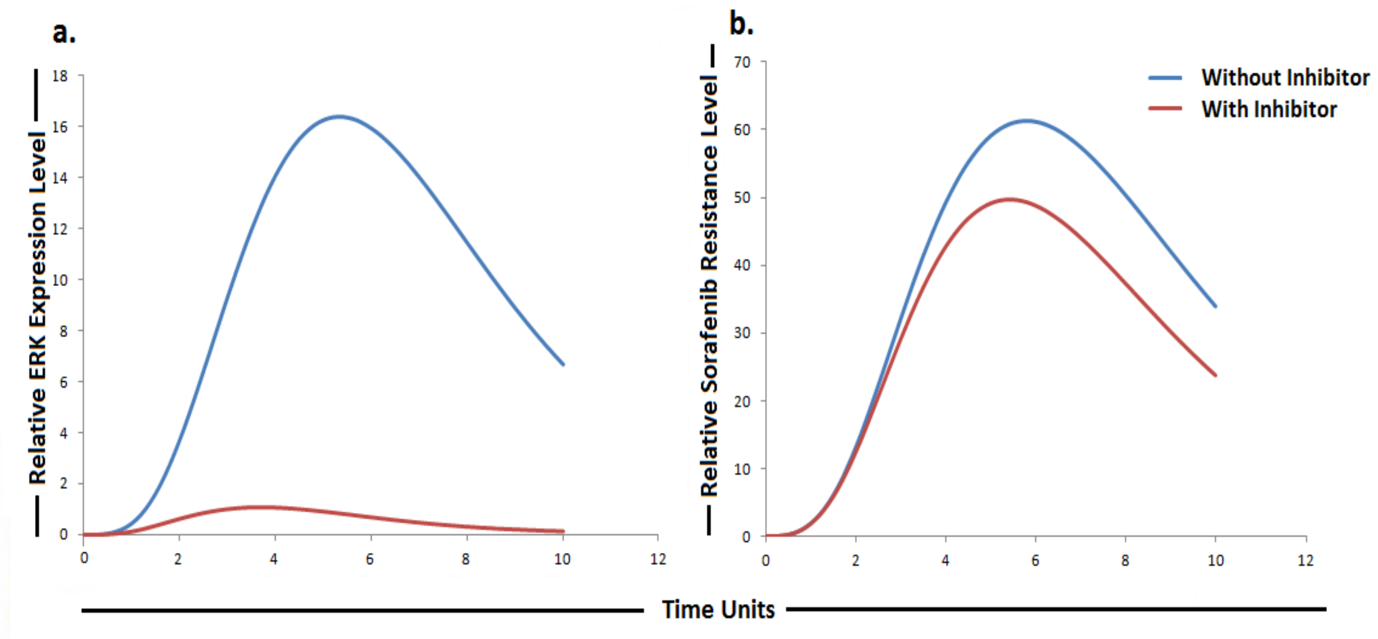

Figure S1. Simulation of relative ERK expression level (a) and relative sorafenib resistance levels (b) with and without ERK inhibitor. Time units are represented on x-axis with relative levels on y-axis

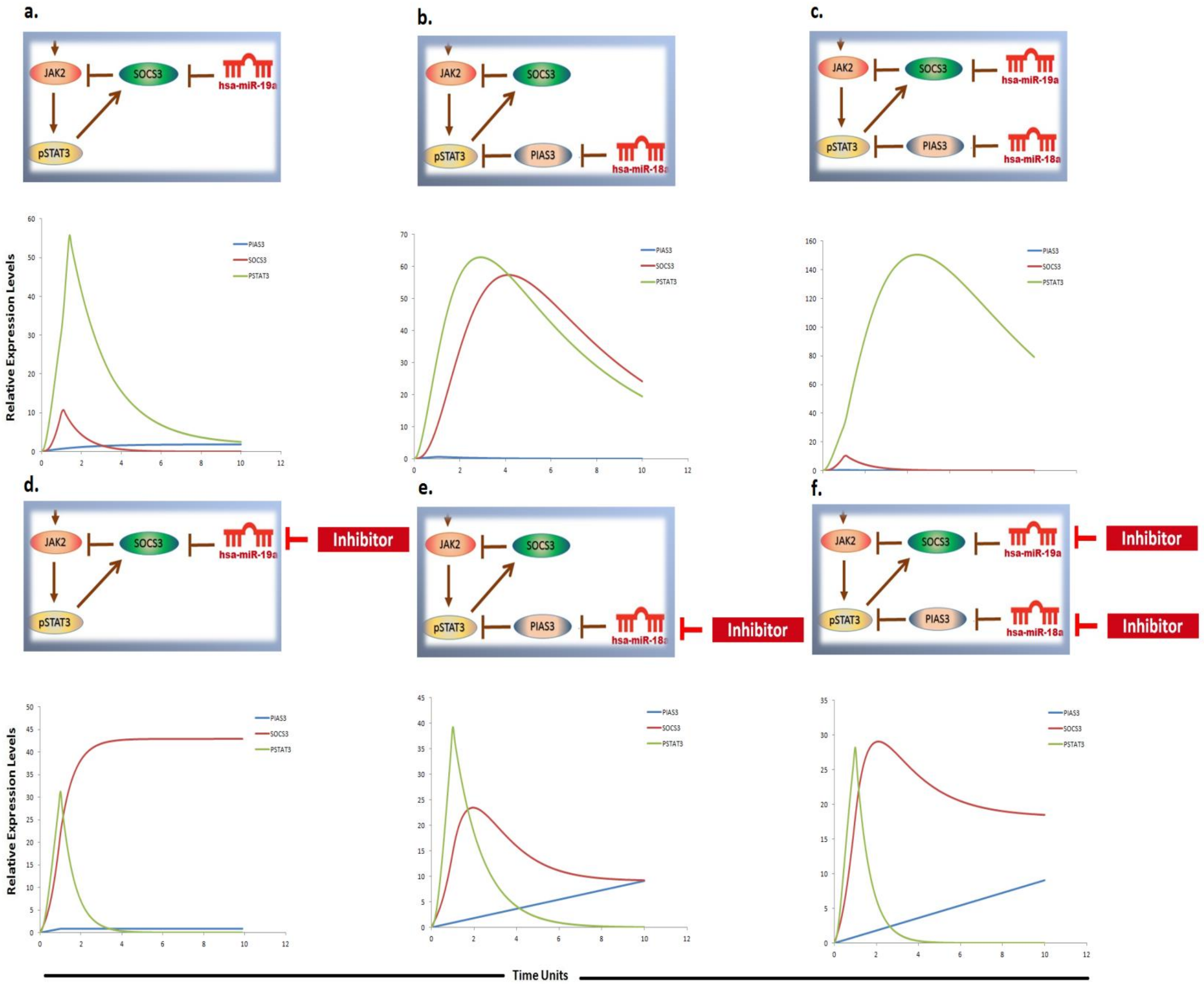

Figure S2. Simulation of SOCS3, PIAS3 and STAT3 in the presence of miR-19a only (a), in the presence of miR-18a only (b), in the presence of both miR-19a and miR18a (c), in the presence of miR-19a inhibitor only (d), in the presence of miR-18a inhibitor only (e), and in the presence of both miR-19a inhibitor and miR-18a inhibitor (f). Time units are represented on x-axis with relative expression levels of SOCS3, PIAS3 and STAT3 on y-axis. Relative levels of SOCS3, PIAS3 and STAT3 are shown in red, blue and green lines respectively.
